# A Dimension Reduction Approach for Energy Landscape: Identifying Intermediate States in Metabolism‐EMT Network

**DOI:** 10.1002/advs.202003133

**Published:** 2021-03-18

**Authors:** Xin Kang, Chunhe Li

**Affiliations:** ^1^ School of Mathematical Sciences Fudan University Shanghai 200433 China; ^2^ Shanghai Center for Mathematical Sciences Fudan University Shanghai 200433 China; ^3^ Institute of Science and Technology for Brain‐Inspired Intelligence Fudan University Shanghai 200433 China

**Keywords:** dimension reduction, energy landscape, epithelial‐mesenchymal transitions, gene regulatory networks, transition paths

## Abstract

Dimension reduction is a challenging problem in complex dynamical systems. Here, a dimension reduction approach of landscape (DRL) for complex dynamical systems is proposed, by mapping a high‐dimensional system on a low‐dimensional energy landscape. The DRL approach is applied to three biological networks, which validates that new reduced dimensions preserve the major information of stability and transition of original high‐dimensional systems. The consistency of barrier heights calculated from the low‐dimensional landscape and transition actions calculated from the high‐dimensional system further shows that the landscape after dimension reduction can quantify the global stability of the system. The epithelial‐mesenchymal transition (EMT) and abnormal metabolism are two hallmarks of cancer. With the DRL approach, a quadrastable landscape for metabolism‐EMT network is identified, including epithelial (E), abnormal metabolic (A), hybrid E/M (H), and mesenchymal (M) cell states. The quantified energy landscape and kinetic transition paths suggest that for the EMT process, the cells at E state need to first change their metabolism, then enter the M state. The work proposes a general framework for the dimension reduction of a stochastic dynamical system, and advances the mechanistic understanding of the underlying relationship between EMT and cellular metabolism.

## Graphical abstract

Dimension reduction is a challenging problem in complex dynamical systems. An effective dimension reduction approach for a dynamical system is proposed based on the energy landscape theory, which provides a general way to quantify the global and stochastic dynamics of dynamical networks. This study also sheds light on the mechanism of the underlying relationship between EMT and cellular metabolism.

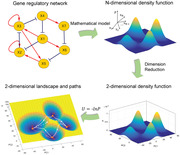

## 1 Introduction

Many physical and biological systems are described by high‐dimensional dynamical systems. Since humans are visual learners, it is important to display the system in an intuitive way, that is, in low‐dimensional space, and study the underlying structural characteristics of the dynamical systems. Therefore, how to perform the dimension reduction is a critical and challenging problem in complex dynamical systems. So far, there are many dimension reduction approaches from a data‐driven perspective, which map samples from a high‐dimensional space to a low‐dimensional space by a linear or nonlinear mapping, such as principal component analysis (PCA),^[^ ^1^ ^]^ linear discriminant analysis (LDA),^[^ ^2^ ^]^ local Linear embedding (LLE),^[^ ^3^ ^]^ and t‐distributed stochastic neighbor embedding (t‐SNE).^[^ ^4^ ^]^ Recently, Pearce et al. proposed a data‐driven dimension reduction approach to infer transition dynamics based on static protein data.^[^ ^5^ ^]^ However, it remains challenging to achieve the dimension reduction for a high‐dimensional dynamical model, which makes it urgent to develop some model‐driven dimension reduction approaches.

The Waddington epigenetic landscape is a classic metaphor for cellular differentiation in biology.^[^ ^6^ ^]^ Recently, the energy landscape theory has been used to study global stability and transition dynamics for some biological systems, for example, high‐dimensional gene regulatory networks.^[^ ^7^, ^8^, ^9^, ^10^, ^11^, ^12^, ^13^, ^14^, ^15^, ^16^ ^]^ An advantage of using landscape theory is that it provides a route to explore the global properties rather than local properties for stable cell states in cellular systems. Secondly, from the landscape theory, it is natural to consider the effects of gene expression fluctuations, which have proven critical in biological networks.^[^ ^17^, ^18^ ^]^ For example, in our previous work, we used a self‐consistent approximation approach to calculate probability distribution and corresponding potential energy landscape for a 52D system, which enables us to study stochastic transition dynamics and global stability of embryonic stem cell in development.^[^ ^7^ ^]^

However, a remaining issue is how to reduce the dimension of the system and study the underlying structure of the energy landscape, which is usually defined in a high‐dimensional space. For example, it is challenging to study the global stability of attractors and transition dynamics on a high‐dimensional landscape. In our previous works,^[^ ^7^, ^8^, ^19^, ^20^ ^]^ to acquire a low‐dimensional landscape, we selected key variables and projected the high‐dimensional landscape to the corresponding 2D space. As such, we can obtain a 2D landscape. One limitation of such dimension reduction approach is that it may lose information for other dimensions, and it is necessary to quantify the results using different pairs of coordinates to see the picture involving more components. Therefore, how to develop a model‐driven dimension reduction approach based on the landscape theory is a crucial issue in studying high‐dimensional dynamical systems.

Here, driven by the data‐based PCA approach, we proposed a model‐based dimension reduction approach of the landscape (DRL) for high‐dimensional systems. We projected a high‐dimensional landscape (corresponding to a probability density function) to a new coordinate system where the first and second coordinates have the top two largest variances of the density function. First, we applied this approach to a general two‐variable model and validated the effectiveness of the DRL approach. Then we applied the DRL approach to two known important gene regulatory networks: mouse embryonic stem cell (MESC) network with 15 nodes^[^ ^20^ ^]^ and human embryonic stem cell (HESC) network with 52 nodes.^[^ ^7^ ^]^ We used the truncated moment equation (TME) method to estimate the probability density functions of systems,^[^ ^19^ ^]^ and reduced the probability density functions to two dimensions with the top two largest variances: PC1 and PC2. In both cases, we showed that the potential barrier heights based on landscape topography from new low‐dimensional system (after dimension reduction) are correlated with transition actions (based on original high‐dimensional space) quantifying the transition feasibility between different cell types. This shows that the landscape in reduced dimensions preserves major information of stability for original high‐dimensional systems.

Epithelial‐mesenchymal transition (EMT) and abnormal metabolism are two hallmarks of cancer.^[^ ^21^, ^22^ ^]^ Next, we applied the DRL approach to a metabolism‐EMT network.^[^ ^19^, ^23^ ^]^ Tumorigenesis depends on the reprogramming of cellular metabolism. In the presence of oxygen, cancer cells often use glycolysis to produce energy, whereas normal cells use glucose for oxidative phosphorylation (OXPHOS). This phenomenon is known as the Warburg effect.^[^ ^24^ ^]^ The EMT plays a critical role in wound healing, organ fibrosis, and the initiation of cancer metastasis.^[^ ^25^ ^]^ Studies have shown that there is an intermediate state H (hybrid E/M cell state) of EMT.^[^ ^26^, ^27^, ^28^, ^29^, ^30^ ^]^ Interestingly, our previous work identified an intermediate abnormal metabolic cell state (A).^[^ ^19^ ^]^ However, whether H state and A state can coexist has not been resolved. Using the DRL approach, we found that the two stable states, H and A, can coexist with a wide range of parameter regions. Based on the TME and DRL approach, we obtained the 2D potential landscape of the metabolism‐EMT model. We identified four stable state attractors on the landscape including epithelial cell state (E), abnormal metabolic cell state (A), hybrid E/M cell state (H), and mesenchymal cell state (M). We further calculated the minimum action paths (MAPs) of the system to quantify the transitions among different cell states. We found that for EMT the cells at E state tend to first change their metabolism, then enter the M state, and for mesenchymal‐epithelial transition (MET), the cells at M state are likely first entering the H state, then reaching the E state. Through global sensitivity analysis, we identified some key components in the network that control EMT. Our work proposes a general way for dimension reduction of energy landscape for a stochastic dynamical system, and promotes our understanding for the mechanisms of the interplay between EMT and metabolism.

## 2 Results

### 2.1 Mathematical Model of Gene Regulatory Networks

In this study, we used a few examples of dynamical systems to demonstrate the effectiveness of our DRL approach. We will first use a general two‐variable model to illustrate the basic idea of our approach. Then we will focus on some specific gene regulatory systems with multistability, which have been extensively studied to model biological systems, including the MESC network,^[^ ^20^ ^]^ the HESC network^[^ ^7^ ^]^ and the metabolism‐EMT network.^[^ ^19^ ^]^ Since our approach starts with a dynamical model, we will first introduce the general form of the dynamical model we are working on. We started from the topology of the networks, and then constructed models of ordinary differential equations (ODEs) to describe the time evolution of levels of different components. In biological models, one often uses Hill functions to describe the activation or inhibition regulations, then the ODEs have the form as:

(1) $$f_{i}\left(x\right)=\frac{dx_{i}}{dt}=\sum_{j=1}^{N}\frac{A_{ji}\times x_{j}^{n}}{S_{ji}^{n}+x_{j}^{n}}+\sum_{j=1}^{N}\frac{B_{ji}\times S_{ji}^{n}}{S_{ji}^{n}+x_{j}^{n}}-k\times x_{i}$$

where $f_{i}$ is the driving force of the system, $x_{i}$ (i=1,…,N) represents the level of component i, k represents the basal degradation rate of $x_{i}$, $S_{ji}$ represents the threshold of a sigmoidal function, and n is the Hill coefficient, which determines the steepness of the sigmoidal function. In addition, A and B are the interaction matrix for the activation and inhibition, respectively. $A_{ji}$ measures the strength of the activation of j on i, and when $A_{ji}$ is equal to zero, it means that there is no activation from component j to component i. $B_{ji}$ measures the strength of the inhibition of j on i, and when $B_{ji}$ is equal to zero, it means that there is no inhibition from component j to component i.

To investigate the stochastic dynamics of the system, we added an external noise to the system. Here, we assumed the Gaussian white noise Γ(t) as the external noise.^[^ ^31^, ^32^, ^33^ ^]^ The equation describing the dynamics of different components changes to a Langevin equation:

(2) $$\dot{x}\left(t\right)=f\left(x\right)+\Gamma \left(t\right)$$

where $x\left(t\right)={\left(x_{1}\left(t\right),x_{2}\left(t\right),\ldots ,x_{N}\left(t\right)\right)}^{T}$, f(x) is a vector composed of $f_{i}\left(x\right)$, i=1,…,N.

In this way, we have a stochastic dynamical model with a general form as in Equation (2). Our following examples all start from the modelling form as in Equations (1) and (2). However, we would like to stress that the DRL approach does not depend on specific form of f(x) and can be applied to a general dynamical system with a general form of f(x).

### 2.2 Dimension Reduction of Landscape (DRL)

Equation (2) provides a stochastic description of a dynamical system. We can obtain corresponding Fokker–Planck equation (FPE, or diffusion equation) of Equation (2), which describes the time evolution of the probability density function of variables. Recent works showed that the Waddington epigenetic landscape metaphor has a rigorous mathematical and chemical kinetic foundation,^[^ ^15^, ^16^, ^34^ ^]^ with the landscape defined as $U=-lnP_{SS}$. Here $P_{ss}$ is steady state probability distribution. Therefore, if we can solve the FPE to obtain the probability distribution of the system, then we have a way to quantify the energy landscape. However, it is challenging to solve the FPE directly for a high‐dimensional system. Here, we resort the moment equation based approach, rather than solve FPE directly. Previously, we have developed a TME method based on the idea of moment equations and Gaussian approximation to approximate the probability density function p(x) of a high‐dimensional system^[^ ^8^, ^19^ ^]^ (see Section 4 for details of the TME method for calculating high‐dimensional probability distribution). Simply speaking, we approximate the density function of a multistable system with M stable points as the weighted sum of M Gaussian distributions, which are determined by solving moment equations.^[^ ^19^, ^35^, ^36^ ^]^ The probability density function takes the form:

(3) $$p\left(x\right)=\sum_{j=1}^{M}\phi^{j}p^{j}\left(x\right)$$

where x is the expression level of a component, $p^{j}\left(\cdot \right)$ is the density function corresponding to the jth stable state, and $\phi^{j}$ is the weight of the corresponding stable state.

However, a remaining issue is how to visualize and analyze a high‐dimensional landscape. Here, we aim to develop an approach to reduce the dimensionality of a high‐dimensional landscape. In our previous works, we selected two variables, projected the density function to a 2D state space by integrating out the other variables, that is, for an N‐dimensional system, we only took the joint distribution of the two of these variables ($P_{SS}$) and obtained the 2D landscape by $U=-lnP_{SS}$ (U is the potential energy and $P_{SS}$ is the steady state probability distribution).^[^ ^7^, ^8^, ^10^ ^]^ Here, we proposed an approach based on the idea of data‐driven PCA to reduce the dimensionality of a high‐dimensional landscape. From the perspective of information theory, the amount of information is equivalent to the amount of uncertainty. The direction with a large variance of the density function is exactly the direction with a large fluctuation, so the uncertainty and the amount of information in this direction is large. Therefore, to preserve more information after dimension reduction, we need to choose directions with large variances as a new coordinate system. For example, if we aim to reduce the dimensionality of the landscape to two dimensions, we should choose the two directions with the top two largest variance of the density function as a new coordinate system. Namely, we can project the system variable to a new coordinate system such that the first coordinate corresponds to the direction with the largest variance of the density function, and the second coordinate corresponds to the direction with the second largest variance of the density function. Then we can calculate the joint density function of the variables in new coordinates and calculate corresponding landscape in reduced low‐dimensional system.

Specifically, for a dynamical system (e.g., gene regulatory networks with multistability), we can acquire the probability density function p(x) of the variables (e.g., the expression level of genes) by the TME method (please see Section 4 for the details of TME method).^[^ ^19^ ^]^ The expectation and covariance of the random variable X in p(x) can be calculated by $\mu =\sum_{j=1}^{M}\phi^{j}\mu^{j}$ and $\Sigma =\sum_{j=1}^{M}\phi^{j}\left(\Sigma^{j}+\mu^{j}{\left(\mu^{j}\right)}^{T}\right)-\mu \mu^{T}$, where M is the total number of stable steady states, and $\mu^{j}$ and $\Sigma^{j}$ are the expectation vector and invertible covariance matrix of $X^{j}$ (corresponding to the jth stable state, see Methods for details), respectively. Based on the idea of PCA, we do eigenvalue decomposition to the covariance matrix, and the two eigenvectors with the top two largest eigenvalues correspond to the two directions with the largest amount of information. We use $W_{2}=\left(w_{1},w_{2}\right)$ to denote the eigenvectors corresponding to the top two largest eigenvalues of Σ, and these two eigenvectors represent the two directions with the top two largest variance. Then, we can project original high‐dimensional system to these two directions. Let $Z_{2}=W_{2}^{T}X$ be the random variable after projection and the joint density function of $Z_{2}$ in 2D space can be expressed by:

(4) $$p_{z_{2}}\left(z_{2}\right)=\sum_{j=1}^{M}\phi^{j}p_{z_{2}}^{j}\left(z_{2}\right)$$

where $p_{z_{2}}^{j}\left(\cdot \right)\left(j=1,\ldots ,M\right)$ is a multi‐dimensional normal distribution, with the expectation vector being the vector composed of the first two components of $W^{T}\mu_{j}$ and the covariance matrix being the matrix composed of the first 2×2 components of $W^{T}\Sigma^{j}W$.

In this way, the variables in the new coordinate system (independent on each other), as new comprehensive indexes, are the linear combination of all the variables in the original system, which preserve as much information about original variables as possible (see Section 4 for details of the DRL approach). Then, the 2D landscape after dimension reduction can be calculated by $U_{2}=-\ln p_{z_{2}}\left(z_{2}\right)$.^[^ ^7^, ^8^, ^10^ ^]^

### 2.3 Dimension Reduction of Landscape for Synthetic Gene Networks

**Figure 1:**
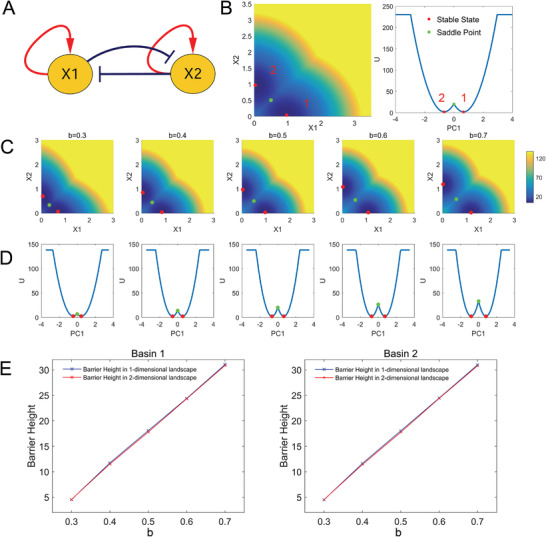
Dimensional reduction for a two‐variable model. A) The network of the MISA model including two nodes. B) Landscapes, stable points, and saddle points shown in original 2D space and 1D space (PC1) after dimension reduction. The contribution rate of PC1 is 95.81%. C) Landscapes, stable points, and saddle points change with parameter b shown in original coordinates. D) Landscapes, stable points, and saddle points change with parameter b shown in PC1 coordinate after dimension reduction. E) The comparisons of barrier heights of each basin changing with parameter b between original 2D landscape and 1D landscape after dimension reduction.

To illustrate the general idea of the DRL approach (the overview of the DRL approach is shown in Figure S1, Supporting Information), we first studied a simplified two‐variable model called mutual inhibition self‐activation (MISA) model, which has been commonly studied as a motif for cell fate decision making.^[^ ^37^ ^]^ Based on the network in **Figure** **1**A, we constructed the ODE model (Equation (S5), Supporting Information, simplified version of Equation (1)). The parameters in this model are set as: a=0.5, b=0.5, S=0.5, n=4, k=1.^[^ ^37^ ^]^ Then we obtained the 1D potential landscape with the DRL approach. Figure 1B shows the landscape in the original coordinates (2D space) and the landscape after dimension reduction (1D space). We see from Figure 1B that on the landscape after dimension reduction (Figure 1B, right), the stable points still maintain their property of being located at the minimum of landscape, and for the saddle point (unstable point), only the non‐convergent direction is retained after dimension reduction, but the property of instability is preserved. To verify whether the landscape after dimension reduction can preserve the global stability (relative stability) of the original system, we changed the parameter b (inhibition constant) and calculated the barrier heights in different coordinate spaces. The barrier height is defined as $BH=|U_{saddle}-U_{stable}|$. From Figure 1C,D, we see that, as the parameter b increases, the corresponding barrier height of each basin increases for both 2D (before dimension reduction) and 1D (after dimension reduction) landscape. We also further showed the quantitative comparisons of barrier height for 2D and 1D landscape (Figure 1E). We see a remarkable consistency for the barrier height calculated from 2D and 1D landscape, demonstrated by the two lines being almost the same (Figure 1E). These results demonstrate that the landscape after dimension reduction preserves the major information on the global stability and transitions for the original system.

To verify the effect of the TME approach on approximating a landscape (probability density), we compared the landscape generated by Langevin simulation method with our TME method (Figure S2, Supporting Information). Since by Langevin simulations the landscape of the system can be acquired directly by the statistics of the long time trajectories, we treated the landscape obtained by Langevin simulations as the solution of the landscape.^[^ ^7^, ^19^ ^]^ Then we used the relative error between the two landscapes to measure the deviation of the landscape calculated by the TME approach from that by Langevin simulations. The relative error is defined as:

(5) $$Error=\sum_{ij}\frac{|U_{ij}^{TME}-U_{ij}^{Simulation}|}{U_{ij}^{Simulation}}$$

From Figure S2, Supporting Information, we found that the landscape obtained by the TME approach and the landscape obtained by Langevin simulations are very close as parameter b varies (Figure S2B,C, Supporting Information). The relative error for landscape estimation of the TME approach is less than 5% as parameter b varies (Figure S2D, Supporting Information). Also the barrier heights on the landscape obtained by the TME approach and the barrier heights obtained by Langevin simulations have a correlation coefficient over 0.99 (Figure S2E, Supporting Information). These results support that the TME approach provides a relatively accurate way to estimate the probability density and energy landscape of a dynamical system.

To see how the DRL approach works for a gene network including more stable states, we studied a synthetic multistable gene network (Figure S3A, Supporting Information). In this model, as we increase the number of positive feedbacks, we can have increasing number of stable states. For example, as we use four nodes with self‐activations, we obtained seven stable states (Table S1 and Figure S3B,C, Supporting Information). We showed the landscape of this system with 7 stable states in 3D and 2D landscape by the DRL approach (Figure S3B,C, Supporting Information).

To verify whether the landscape after dimension reduction can preserve the quantitative properties of stability for the original system, we need to make a comparison for the system before and after dimension reduction. A direct way is to compare barrier height for the landscape before and after dimension reduction. We did this for relative lower‐dimensional system, that is, for the above two‐variable MISA model. However, how to calculate the barrier height of a general high‐dimensional system still warrants explorations, since it requires the calculation of all stable points and unstable points for a system with many stable states. Here, we used an alternative way to show the effectiveness of the DRL approach. We calculated the potential energy of different stable states as parameter a (activation constant) changes before and after dimension reduction (Figure S3D, Supporting Information). We see that the potential energy U at different stable states has similar trend as a changes for original 4D and reduced 3D or 2D landscape. This is also true for the relative potential barrier height when we took stable state 1 and 4, as well as stable state 4 and 7 as examples (Figure S3E, Supporting Information), which indicates that the major information of stability for stable states is preserved. These results support that the DRL approach has the potential to be applied to the system with many stable states.

### 2.4 Potential Landscape in Reduced Dimensions for the Mouse Embryonic Stem Cell (MESC) Network

**Figure 2:**
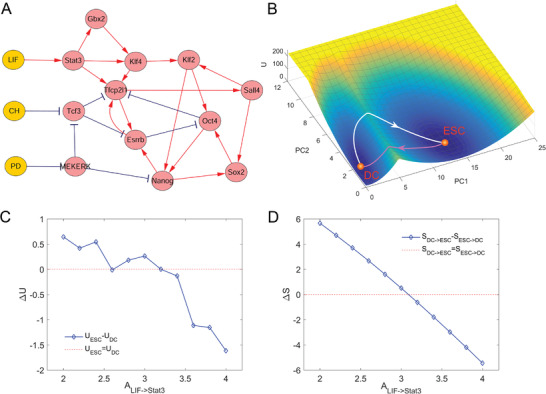
Landscape in reduced dimensions for a MESC network. A) The network of the MESC model including 15 nodes and 26 interaction links (18 activations and 8 inhibitions). Red arrows represent activation and blue perpendicular bars represent inhibition. Orange nodes represent input signals, and red nodes represent genes. B) Landscape and transition paths shown in PC1‐PC2 coordinate. The contribution rates of PC1 and PC2 are 95.52% and 1.15%, respectively. The blue region represents higher probability or lower potential and the yellow region indicates lower probability or higher potential. The white line represents the transition path from DC state to ESC state, and the magenta line represents the transition path from ESC state to DC state. C) The relation between the regulation strength of LIF on Stat3 ($A_{\mathrm{LIF}\to \mathrm{Stat}3}$) and the relative barrier heights (difference between $U_{ESC}$ and $U_{DC}$). D) The relation between the regulation strength of LIF on Stat3 ($A_{\mathrm{LIF}\to \mathrm{Stat}3}$) and the relative transition actions (difference between $S_{DC->ESC}$ and $S_{ESC->DC}$). ESC, embryonic stem cell state; DC, differentiated cell state.

In the following sections, we will apply the DRL approach to three realistic biological models with multistability. First, we studied the MESC differentiation model with the DRL approach. The heterogeneity and multistable property in stem cell differentiation have been explored in previous work.^[^ ^20^ ^]^ This network includes 26 interactions (regulations) and 15 components (12 genes and 3 signals)^[^ ^20^, ^38^ ^]^ (**Figure** **2**A and Table S2, Supporting Information). The three extra‐cellular signals are cytokine leukemia inhibitory factor (LIF) and two selective inhibitors, including glycogen synthase kinase 3 (CH) and mitogen‐activated protein kinase kinase (PD).^[^ ^39^ ^]^ Stat3, Gbx2, Klf4, Tcf3, Tfcp2l1, Esrrb, MEKERK, and Nanog are direct transcriptional targets of LIF, CH, and PD.^[^ ^40^, ^41^, ^42^, ^43^, ^44^, ^45^ ^]^ The other regulations among genes are inferred from experimental data of mouse embryonic stem cells.^[^ ^38^ ^]^

Based on this network, we wrote down the ODE models,^[^ ^20^ ^]^ and added a constant $g_{0}=0.01$ to Equation (S5), Supporting Information as the basic synthetic rate. The parameters in this model are set as: a=3.8, b=0.4, S=2.9, n=4, k=1.^[^ ^20^ ^]^ Then we obtained the potential landscape with the DRL approach. Table S3, Supporting Information shows the new coordinates after dimension reduction. After the dimension reduction, the coordinate of a point in the 2D space can be represented by ${\left(z_{1},z_{2}\right)}^{T}$, where $z_{i}=w_{i}^{T}x$, $w_{1}$ and $w_{2}$ are the directions with the top two largest variances, and x is the coordinate of the corresponding point in the original high‐dimensional space. The contribution rate (the ratio of the ith eigenvalue to the sum of all eigenvalues, which represents the proportion of the original information carried by the current principal component) of the PC1 coordinate is 95.52%, and the top five genes having major contributions to PC1 include OCT4, Sox2, Nanog, Esrrb, and Tfcp2l1, which are exactly marker genes of mouse embryonic stem cells. This indicates that the DRL approach preserves the information of stability successfully for this network. Therefore, we can distinguish the two stable states of the system by the level of PC1. The embryonic stem cell (ESC) state has a high PC1 level, while the differentiated cell (DC) state has a low PC1 level.

For the MESC network, we also compared the landscape calculated by the TME approach with that obtained by Langevin simulations (Figure S4, Supporting Information). We see that the barrier height of each basin obtained by the TME method has a similar trend as the barrier height obtained by Langevin simulations as the parameter b (inhibition constant) increases, and the correlation coefficients of them for each basin are over 0.9. These results support that the TME and DRL approaches can approximate the probability density well for a high‐dimensional system.

With the DRL approach, we obtained the landscape of the MESC system. Figure 2B shows the 2D landscape in PC1 and PC2 coordinates, where two basins (attractors) appear on the landscape, representing two cell states, respectively. We acquired the kinetic transition paths between the two cell states by minimizing the transition actions, which are also called minimum action paths (MAPs) (see Section 4 for details of MAPs).^[^ ^46^, ^47^ ^]^ Since the transition paths and transition actions are calculated in original system, the transition paths are in 12D space. We can project the transition paths onto the landscape after dimension reduction. For an N‐dimensional path with K points denoted by $l_{i}$ (i=1,…,K), the point on the paths after dimension reduction is $h_{i}=\left(w_{1}^{T},w_{2}^{T}\right)l_{i}$ (i=1,…,K). Figure 2B also shows the kinetic transition paths shown in PC1 and PC2 coordinates, where the magenta path represents differentiation (from ESC to DC) and the white path represents reprogramming (from DC to ESC).

As discussed above, how to calculate the barrier height of a high‐dimensional system remains challenging. To see whether the DRL can preserve the major information on stability of original high‐dimensional system, here we resort to an alternative way to characterize high‐dimensional system, which is to calculate the transition actions, since the transition action and barrier height have similar physical meaning for quantifying the transition feasibility from one attractor to another.^[^ ^37^ ^]^ We explored the correlation between the transition action from one attractor to the other (in original high‐dimensional system) and corresponding potential barrier height (in low‐dimensional system after dimension reduction). We defined relative barrier height $RBH=U_{ESC}-U_{DC}$ and relative transition action $\Delta S=S_{DC->ESC}-S_{ESC->DC}$ to quantify the transition feasibility from ESC state to DC state. The results are shown in Figure 2C,D. We see that as the regulation strength LIF → Stat3 increases, the RBH decreases. This indicates that the enhancement of the regulation LIF → Stat3 in the network leads to a more stable ESC state, making it easier for the transition from DC state to ESC state. Meanwhile, as the regulation strength LIF → Stat3 increases, the ΔS also decreases, which also leads to an easier transition from DC state to ESC state. Therefore, the relative barrier heights and relative transition actions have a similar trend as the link strength changes. This indicates that the landscape after dimension reduction preserves major information on relative stability and transition of the original high‐dimensional system.

### 2.5 Potential Landscape in Reduced Dimensions for the Human Embryonic Stem Cell (HESC) Network

**Figure 3:**
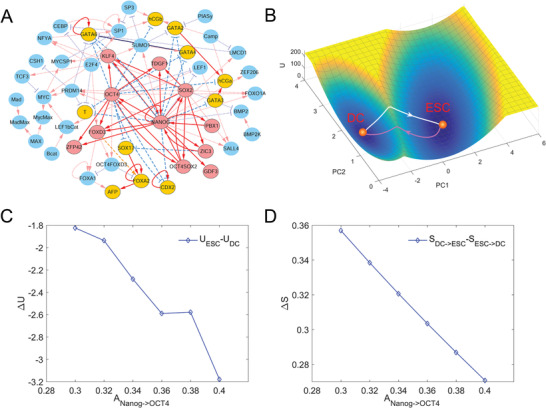
Landscape in reduced dimensions for a HESC network. A) The network of the HESC model including 52 gene nodes and their interactions (red arrows represent activation and blue perpendicular bars represent inhibition). Red nodes represent 11 marker genes for the pluripotent stem cell state, orange nodes represent 11 marker genes for the differentiation cell state, and cyan nodes represent other genes. The solid links represent the links between marker genes, and the dashed links represent the other links. B) Landscape and transition paths shown in PC1‐PC2 coordinate. The blue region represents higher probability or lower potential and the yellow region indicates lower probability or higher potential. The contribution rates of PC1 and PC2 are 16.91% and 4.17%, respectively. The white line represents the transition path from DC state to ESC state, and the magenta line represents the transition path from ESC state to DC state. C) The relation between the regulation strength of NANOG on OCT4 ($A_{\mathrm{NANOG}\to \mathrm{OCT}4}$) and the relative barrier heights (difference between $U_{ESC}$ and $U_{DC}$). D) The relation between the regulation strength of NANOG on OCT4 ($A_{\mathrm{NANOG}\to \mathrm{OCT}4}$) and the relative transition actions (difference between $S_{DC->ESC}$ and $S_{ESC->DC}$). ESC, embryonic stem cell state; DC, differentiated cell state.

To see whether our approach works in the models with higher dimensions, we applied our DRL approach to a HESC developmental network composed of 52 genes.^[^ ^7^ ^]^ The network topology is shown in **Figure** **3**A (Table S4, Supporting Information). We wrote down the ODEs and obtained the 2D landscape with the DRL approach. The parameters in this model are set as: a=0.37, b=0.5, S=0.5, n=3, k=1.^[^ ^7^ ^]^ This system exhibits bistable state for ESC and DC cells. Table S5/, Supporting Information shows the new coordinates of this system after dimension reduction. Number 1–11 of the genes are stem cell marker genes, and number 12‐22 of the genes are differentiation marker genes. The coordinates of a point in PC1‐PC2 plane can be represented by ${\left(z_{1},z_{2}\right)}^{T}={\left(w_{1}^{T}x,w_{2}^{T}x\right)}^{T}$, where $w_{1}$ and $w_{2}$ are the directions with the top two largest variance and x is the coordinate of the corresponding point in the original high‐dimensional space. It can be seen from Table S5, Supporting Information that the expression levels of stem cell marker genes are positively correlated with PC1, while the expression levels of differentiation marker genes are negatively correlated with PC1. Therefore, the two stable states can be distinguished by PC1, that is, the ESC state has a high PC1 level, while the DC state has a low PC1 level.

Further, we obtained the landscape and the MAPs for the HESC network (Figure 3B). For this network, we changed the regulation strength of the link Nanog → OCT4 to investigate the correlation between the transition actions and the corresponding potential barrier heights. We also defined relative barrier height $RBH=U_{ESC}-U_{DC}$ and relative transition action $\Delta S=S_{DC->ESC}-S_{ESC->DC}$ to quantify the transition feasibility from the ESC state to the DC state as before. From Figure 3C,D, we see that as the regulation strength of the link Nanog → OCT4 increases, the relative barrier height RBH and relative transition action ΔS both decrease, which indicates that the enhancement of the regulation strength of Nanog → OCT4 in the network leads to less transition action from DC state to ESC state, or more transition actions from ESC state to DC state. These results suggest that the potential barrier height (low‐dimensional system) is a good approximation of transition actions (original high‐dimensional system), and the landscape with reduced dimensions preserves the information of stability and transition of the original high‐dimensional system.

### 2.6 Potential Landscape in Reduced Dimensions for the Metabolism‐EMT Network

**Figure 4:**
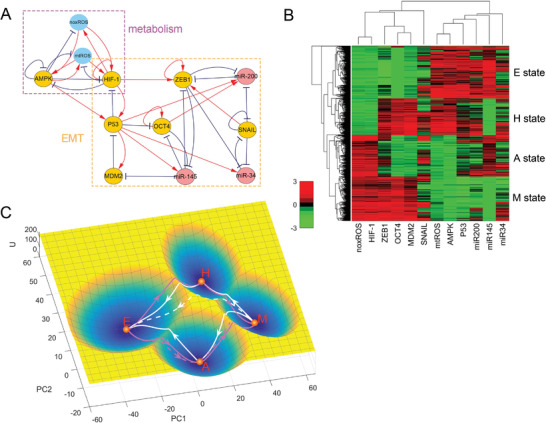
Stable states for a metabolism‐EMT model. A) The network of the metabolism‐EMT model including 12 nodes and 39 interaction links (18 activations and 21 inhibitions). Red arrows represent activation and blue perpendicular bars represent inhibition. Orange nodes represent genes, cyan nodes represent metabolites, and pink nodes represent microRNAs. B) Hierarchical clustering analysis of the stable states from 1000 parameter sets. Each row represents a stable state, and each column represents a gene (or metabolite, microRNA). The stable states can be clustered into four main clusters, which characterize epithelial cell (E), abnormal metabolic cell (A), hybrid E/M cell (H), and mesenchymal cell (M) states, respectively. (C) Landscape and paths shown in PC1‐PC2 coordinate. The contribution rates of PC1 and PC2 are 71.36% and 26.88%, respectively. The blue region represents higher probability or lower potential and the yellow region indicates lower probability or higher potential. Magenta lines represent the transition paths from the E state to M state. White lines represent the transition paths from M state to E state. The solid lines represent indirect paths and the dashed lines represent direct paths. E, epithelial cell state; A, abnormal metabolic cell state; H, hybrid E/M cell state; M, mesenchymal cell state.

With the insights derived from above models, we applied our approach to a metabolism‐EMT network, where we aim to explore the interplay between metabolism and EMT.^[^ ^19^, ^23^ ^]^ There are 12 components in the network, including seven genes (AMPK, HIF‐1, P53, ZEB1, OCT4, SNAIL, and MDM2), three microRNAs (miR‐145, miR‐200, and miR‐34), and two metabolites (noxROS and mtROS). AMPK, HIF‐1, mtROS, and noxROS are the core components controlling cellular metabolism. SNAIL, ZEB1, OCT4, MDM2, miR‐145, miR‐200, miR‐34, and P53 are the core components governing the EMT. **Figure** **4**A (Table S6, Supporting Information) shows the network topology of the metabolism‐EMT network. We constructed the model describing the dynamical evolution of the system. Then, we searched parameters in the parameter space based on the biological significance of stable states to determine the parameter values in the ODEs. Since specific parameter values are not available from experiments, we chose one of the parameter sets that can generate biologically significant quadrastable states. That is, one of four stable states represents the mesenchymal state, which has higher expression levels of HIF‐1 and ZEB1, lower expression levels of miR‐145, miR‐200 and miR‐34, and one of four stable states represents the epithelial state, which has lower expression levels of HIF‐1, ZEB1, higher expression levels of miR‐145, miR‐200 and miR‐34 (see Supporting Information for the parameter search algorithm to determine the parameters). A typical parameter set for our model from parameter search algorithm is shown in Table S7, Supporting Information. There are four stable states under this parameter set, which correspond to epithelial (E), abnormal metabolic (A), hybrid E/M (H), and mesenchymal (M) cell states, respectively (see Table S8, Supporting Information for the gene expression levels of the stable steady states).

To verify the robustness of the system against parameter perturbations, we utilized a parameter perturbation approach.^[^ ^23^ ^]^ We randomized the modeling parameter sets and each parameter set is randomly sampled from (75%p, 125%p), where p is the default parameter set in our model. Then, we collected all the stable states and used the clustergram function of MATLAB to implement hierarchical clustering analysis to identify the groups of the stable states. It can be seen from Figure 4B that the stable states form four groups: one is E state, which has lower expression levels of HIF‐1 and ZEB1; one is A state, which has lower expression levels of ZEB1 and higher expression levels of HIF‐1; one is H state, which has lower expression levels of HIF‐1 and higher expression level of ZEB1; and the other one is M state, which has higher expression levels of HIF‐1 and ZEB1 (Figure 4B). The result of parameter perturbation shows that our model is robust against parameter perturbations.

We further obtained the landscape with the DRL approach. Table S9, Supporting Information shows the new coordinates of the metabolism‐EMT model after dimension reduction. We see from Table S9, Supporting Information that the PC1 coordinate is positively correlated with HIF‐1 and ZEB1, while negatively correlated with miR200, miR145, and miR34. Since HIF‐1 is a marker gene of cellular metabolism, ZEB1 is a marker gene of EMT and over‐expression of miR‐145 attenuates EMT,^[^ ^48^ ^]^ we can distinguish E state and M state by PC1. The E state has a low PC1 level, while the M state has a high PC1 level.

### 2.7 Kinetic Transition Paths for the Metabolism‐EMT Network

To quantify the kinetic transitions among these states, we also calculated the MAPs among different cell states. Since the terminal time T is a key parameter for the calculations of the MAPs, larger T can lead to more accurate calculation of transition actions, but can also lead to instabilities.^[^ ^46^ ^]^ It is important to find a suitable T. Therefore, we calculated the transition paths and the corresponding transition actions with different terminal time T. Figure S5, Supporting Information shows the transition actions among the different states. All the transition actions among the stable states decrease with the increase of the termination time T, and when T>120, the transition actions gradually converge. Figure S6, Supporting Information shows the transition paths with different terminal time T in PC1‐PC2 coordinates. It can be seen that the transition paths have a similar pattern when T≥100. Therefore, we chose a larger terminal time T=140 to calculate the transition paths among the different stable states. The 2D landscape and transition paths are displayed in Figure 4C. The direct transition paths from E state to M state and the backward transition paths from M state to E state are not identical. The kinetic paths of the system deviate from the conventionally expected potential gradient paths, which is caused by the non‐gradient force, that is, curl flux.^[^ ^8^, ^13^ ^]^

**Figure 5:**
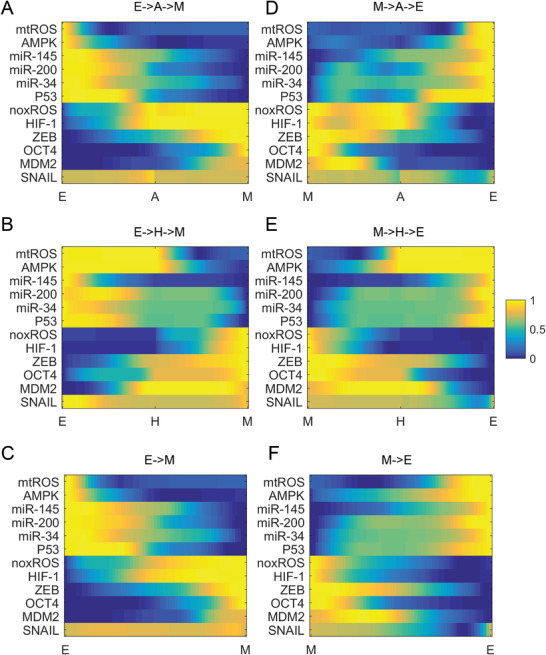
The normalized transition paths for the metabolism‐EMT model when the terminal time T=140. *Y*‐axis represents the 12 genes and *X*‐axis represents the time points along the transition paths. A,B) The indirect transition path from E state to M state shown for 12 components. D,E) The indirect path from M state to E state shown for 12 components. C,F) The direct transition path between E state and M state shown for 12 components. The yellow regions represent higher expression levels and the blue regions indicate lower expression levels. E, epithelial cell state; A, abnormal metabolic cell state; H, hybrid E/M cell state; M, mesenchymal cell state.

Furthermore, in the PC1‐PC2 coordinates, the direct transition path from E state to M state (the magenta dashed line) has a similar pattern as the indirect transition path from E state to A state and then to M state (the magenta solid line), that is, the transition path from E state to M state tends to first go through the A state (Figure 4C). However, the transition path from M state to E state does not coincide with the indirect path M→A→E or M→H→E in the PC1‐PC2 coordinates. We normalized the paths and obtained the result of 12D paths (**Figure** **5**). Figure 5 shows that the direct path from E state to M state is closer to the indirect path E→A→M and the direct path from M state to E state is closer to the indirect path M→H→E. To further verify these results, we calculated the Euclidean distance between the indirect transition paths and the direct transition paths. The Euclidean distance is defined as:

(6) $$Dis=\frac{1}{N}\sum_{i=1}^{K}\sqrt{\sum_{j=1}^{N}{\left(x_{ij}-y_{ij}\right)}^{2}}$$

where x and y are two N‐dimensional paths with K points. Figure S7, Supporting Information shows the changes of the Euclidean distance with the terminal time T. For all terminal time T, the Euclidean distance between the indirect path E→A→M and the direct path E→M is smaller than the distance between E→H→M and E→M. This demonstrates that transition from E state to M state tends to go through the A state, which is consistent with our previous models and experimental data.^[^ ^19^ ^]^ Several experiments have shown that HIF‐1α can promote EMT,^[^ ^49^, ^50^ ^]^ which suggests that cells might arrive at an abnormal metabolic state first during EMT. As for the transition path from M state to E state, it can be seen that when the terminal time T is small, the transition path from M state to E state is more inclined to first go through the A state. On the contrary, when the terminal time T is large, the transition path from M state to E state is more inclined to first go through the H state. Since a large enough time T is important to capture the correct optimal transition path, the transition path is closer to the correct optimal transition path when the terminal time T is large. Therefore, the transition path from M state to E state is more likely to first go through the H state, which is consistent with what we observed from Figure 5.

To identify the robust transition paths for the metabolism‐EMT network, we used the parameter perturbation approach to calculate the stable states and the transition paths. Since larger perturbation will lead to the phase transition, we chose a 5% boundary here. We sampled 100 parameter sets from (95%p, 105%p), where p is the default parameter set in our model. For each parameter set, we calculated the stable states and the transition paths. Then, we collected all stable states and used PCA to reduce the dimensionality of the stable states. The transition paths are also projected onto the corresponding dimensions. Figure S8, Supporting Information shows the stable states and the transition paths after dimension reduction. Although the stable states of different parameter sets are different, they have a similar expression level mode. The stable states can also be divided into four groups (E, A, H, and M), and the most transition paths also follow the similar mode as Figure 4C. This shows that our results of transition paths are robust against parameter perturbations. Only a small number of direct paths from E state to M state are different from Figure 4C, probably because the current terminal time T that we used is not large enough for those systems.

**Figure 6:**
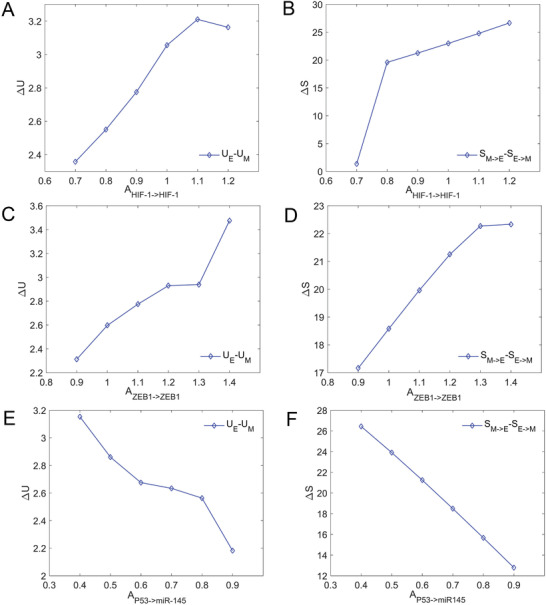
The relative barrier heights (difference between $U_{E}$ and $U_{M}$) and relative transition actions (difference between $S_{E->M}$ and $S_{M->E}$) change as regulatory strengths change. A,C,E) show the relative barrier height changes with the self‐activation strength of HIF1 $A_{\mathrm{HIF}-1\to \mathrm{HIF}-1}$ A), the self‐activation strength of ZEB1 $A_{\mathrm{ZEB}1\to \mathrm{ZEB}1}$ C), and the activation of P53 on miR‐145 $A_{P53\to \mathrm{miR}-145}$ E), individually. B,D,F) show the corresponding trend for transition actions. E, epithelial cell state; A, abnormal metabolic cell state; H, hybrid E/M cell state; M, mesenchymal cell state.

For the metabolism‐EMT network, we also investigated the correlation between the transition actions (original high‐dimensional space) and the potential barrier height (low dimensional space after dimension reduction). For the regulation strength of HIF‐1 → HIF‐1, ZEB1 → ZEB1, and P53 → miR‐145, we calculated relative barrier height ($RBH=U_{E}-U_{M}$) and relative transition action ($\Delta S=S_{M->E}-S_{E->M}$) as these regulation strengths increase, respectively. **Figure** **6** shows the changes of RBH and ΔS are correlated and consistent. This shows that the landscape after dimension reduction can capture the information of stability and transition of the high‐dimensional metabolism‐EMT system.

### 2.8 Global Sensitivity Analysis for the Metabolism‐EMT Network

**Figure 7:**
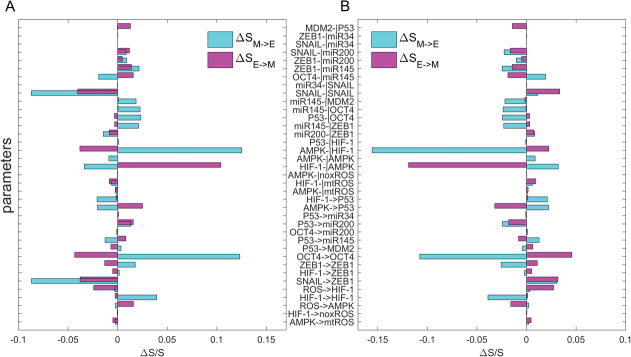
Global sensitivity analysis for the parameters based on transition actions for the metabolism‐EMT model. *Y*‐axis represents the 37 parameters. *X*‐axis represents the percentage of the change of the transition action (S) relative to S with default parameters. Here, $S_{E->M}$ represents the transition action from E state to M state (magenta bars), and $S_{M->E}$ represents the transition action from M to E state (cyan bars). A) Each parameter is increased by 10%, individually. B) Each parameter is decreased by 10%, individually. E, epithelial cell state, M, mesenchymal cell state.

To further study the effects of parameters on the dynamics of the network, we performed a global sensitivity analysis on parameters for the metabolism‐EMT model. We calculated the transition actions between the E state and M state to quantify the feasibility of transitions for EMT and mesenchymal to epithelial transition (MET). We increased or decreased each parameter (specifically we changed the elements of the interaction matrix A and B, that is, $A_{ji}$ and $B_{ji}$, to study the effects of different regulatory strengths on the system) by 10%, individually. Then we calculated how the transition actions between the E state and M state change after these perturbations. **Figure** **7** shows the results of global sensitivity analysis. The links are sorted according to their sensitivities (defined as $▵S_{M\to E}-▵S_{E\to M}$). The most sensitive regulations are shown in Table S10, Supporting Information. It can be seen that HIF‐1 ⊣ AMPK ($B_{\mathrm{HIF}-1⊣\mathrm{AMPK}}$), AMPK ⊣ HIF‐1 ($B_{\mathrm{AMPK}⊣\mathrm{HIF}-1}$) and OCT4 → OCT4 ($A_{\mathrm{OCT}4\to \mathrm{OCT}4}$) are the most sensitive links. Both increasing and decreasing the strength of HIF‐1⊣AMPK have a great influence on the stability of E state and M state. Increasing $B_{\mathrm{HIF}-1⊣\mathrm{AMPK}}$ (decreasing inhibition strength of HIF‐1 ⊣ AMPK) can make E state more stable, while decreasing $B_{\mathrm{HIF}-1⊣\mathrm{AMPK}}$ can make M state more stable. On the contrary, the increase of $B_{\mathrm{AMPK}⊣\mathrm{HIF}-1}$ and $A_{\mathrm{OCT}4\to \mathrm{OCT}4}$ can make M state more stable, while the decrease of $B_{\mathrm{AMPK}⊣\mathrm{HIF}-1}$ and $A_{\mathrm{OCT}4\to \mathrm{OCT}4}$ can make E state more stable. Some predictions agree well with previous experimental studies. For example, the expression level of HIF‐1 is stable only under hypoxia and hypoxia is able to switch on an EMT programme leading cancer cells to acquire a fibroblastoid‐like phenotype and to display a significantly increased invasive propensity.^[^ ^50^ ^]^ Oct4 can activate LEF1/β‐catenin dependent WNT signaling pathway and induce EMT.^[^ ^51^ ^]^

## 3 Discussion

The energy landscape theory has been used to study the global properties and transition dynamics for biological systems.^[^ ^7^, ^8^, ^9^, ^10^, ^12^ ^]^ To visualize and analyze a high‐dimensional landscape, we proposed a dimension reduction approach for landscape. After dimension reduction, the new two coordinates correspond to the two directions with the top two largest variances. We found that the two top PC coordinates after dimension reduction are positively or negatively correlated with marker genes for the three biological networks we studied. Therefore, we can distinguish different stable states by a few top PC coordinates. This also indicates that we can identify the key regulatory factors that govern the behavior of the system by a few top PC coordinates. Besides, the depths of each attractor of the landscape are the quantitative measures of the degrees of stability. We found that the relative barrier heights of the 2D landscape are consistent with the relative transition actions in original high‐dimensional system as parameters vary, which indicates that the landscape after dimension reduction preserves the information of stability and transition of original high‐dimensional systems.

One advantage of using PCs as coordinates for a dynamical system is this way of picking coordinates is completely determined by the properties of system dynamics, which does not require prior knowledge of biological significance for different components (e.g., the information of marker genes in gene networks). Secondly, here we showed three realistic networks including stem cells and cancer related networks, which have been extensively studied, and we already had some knowledge about marker genes of these networks. In a realistic new network, by examining PC coordinates, which are combinations of certain genes, one could potentially infer important factors (genes) from a new network. For example, in Table S3, Supporting Information, a group of genes (rather than one specific gene) with larger weight factor in PC1 could be considered important factors in this network. So, this approach could also be useful for experimentalists to infer critical factors for a new gene network. For example, the synthetic multistable gene networks are very important tools to study multistability and cell fate transition mechanisms.^[^ ^52^, ^53^, ^54^ ^]^ We believe that the proposed DRL approach can be potentially applied to synthetical gene circuits to calibrate model parameters and infer critical factors based on quantitative experimental data.

In three biological networks we studied here, we projected the original high‐dimensional systems onto a 2D landscape (PC1 and PC2) for visualization. However, the DRL approach we proposed here can be generally expanded to include more dimensions after dimension reduction. A question would be how many PC coordinates are needed to characterize the system dynamics. This may be related to the network size and dynamical properties of the specific systems, and can be determined by checking the contribution rate of different PCs. For example, in the MESC network (Section 2.4, Figure 2, and Table S3, Supporting Information), the first two PCs have the total contribution rate of 96.67 (95.52% + 1.15%) (Table S3, Supporting Information). In the EMT network (Section 2.6, Figure 4, and Table S9, Supporting Information), the first two PCs have the total contribution rate of 98.24 (71.36% + 26.88%) (Table S9, Supporting Information). Therefore, for these two cases, the first two PCs have very large contribution rate, and using the first two PCs as coordinates should be a reasonable choice to characterize the stability of the original system. We also noticed that in the HESC network (Section 2.5, Figure 3, and Table S5, Supporting Information), the total contribution rate of the first two PCs is only about 21% (Table S5, Supporting Information). So, in this case, it may be useful to use more PC coordinates.

The DRL approach proposed here based on Gaussian approximation has certain limitations. For example, current approach is based on continuous dynamical system (described by the Fokker–Planck equation). In biology, it is also important to use discrete dynamical system to describe certain biological behavior, for example, RNA copy number or epigenetic regulations.^[^ ^55^, ^56^, ^57^ ^]^ Some approaches based on chemical master equations using discrete form of descriptions for gene and epigenetic regulations have been developed to quantify the energy landscape from different perspectives.^[^ ^12^, ^58^ ^]^ It will be of great interest to combine the Gaussian approximation approach with approaches based on chemical master equations to study the stochastic dynamics of discrete dynamical systems, involving gene and epigenetic regulations.

In this work we focused on multistable systems widely used to model biological networks. We tried different scenarios, including the MESC network with relatively large network size, the EMT network representing a realistic complex biological network with four stable states, and a synthetic gene network generating more stable states (seven stable states). With these examples, we showed that the low‐dimensional system after dimension reduction preserves the major stability and transition properties of original high‐dimensional system. To see how well the DRL approach works in a system including more stable states, we explored a synthetic multistable gene network, which can generate seven stable states. So, for the issue we studied here, the complexity may come from two aspects. One is the dimensionality of the system, and the other comes from the large number of stable states in the system. Although the DRL approach we proposed does not depend on the specific form of dynamical models, it remains an interesting problem to explore how well the DRL approach can be applied to characterize a more general dynamical system, for example, more complex systems with even more stable states and limit cycle oscillation systems.^[^ ^8^, ^59^ ^]^

Abnormal metabolism and EMT are two hallmarks of cancer.^[^ ^21^, ^22^ ^]^ Recent studies have shown that abnormal metabolism in cancer cells can induce EMT through multiple pathological pathways and EMT exacerbates dysregulation of glucose metabolism by some EMT transcriptional factors.^[^ ^60^ ^]^ In this work, we used the DRL approach to investigate the stochastic dynamics of the gene regulatory network for the interplay between metabolism and EMT.^[^ ^19^ ^]^ Here, we found a wide range of parameter regions that can generate four stable states, which correspond to epithelial (E), abnormal metabolic (A), hybrid E/M (H), and mesenchymal (M) cell states, respectively. We further calculated the MAPs of the system to quantify the transition paths among these states, which are treated as the biological paths. The results of the MAPs show that cells prefer to follow a sequential order during EMT or MET. For EMT the cells at E state need to first enter the A state, then enter the M state, and for MET the cells at M state are likely first entering the H state, then reaching the E state. These predictions are partly consistent with experimental data and can be tested in future experiments. By global sensitivity analysis of parameters, we provided some predictions on the key links controlling EMT that are partly consistent with experimental data.

In summary, we proposed an effective approach to reduce the dimension for a dynamical system based on the energy landscape theory, and a general way to quantify the global and stochastic dynamics of gene regulatory networks. Our work advances the mechanistic and quantitative understanding of the underlying relationship between EMT and cellular metabolism.

## 4 Methods

### 4.1 Truncated Moment Equation (TME)

The time evolution of a dynamical system can be described by the the Langevin equation as in Equation (2). Here, $x\left(t\right)=(x_{1}\left(t\right)$, $x_{2}\left(t\right)$, ⋯, $x_{N}{\left(t\right))}^{T}$ represents a stochastic variable vector (e.g., gene expression level), f(x(t)) represents the driving force of the system, and $\Gamma \left(t\right)=(\Gamma_{1}\left(t\right),\Gamma_{2}\left(t\right),\ldots$, $\Gamma_{N}{\left(t\right))}^{T}$ is N‐dimensional independent Gaussian white noise, which means:

(7) $$E[\Gamma_{i}\left(t\right)]=0$$

(8) $$E\left[\Gamma_{i}\left(t\right)\Gamma_{j}\left(t^{\prime }\right)\right]=2d\delta_{ij}\delta_{0}\left(t-t^{\prime }\right)$$

Here, we assume that the noise is homogeneous, and only consider the external noise, so d is the constant diffusion coefficient and

(9) $$\begin{array}{c} \delta_{ij}=\left\{\begin{array}{cc} 1, & i=j\\ 0, & i\neq j \end{array}\right. \end{array}$$

In Equation (8), $\delta_{ij}$ means the noises are independent for different i and j, and $\delta_{0}\left(t-t^{\prime }\right)$ is Dirac Delta function, which means for one variable the noises at different times are independent. The time evolution of this dynamical system is determined by the FPE or probabilistic diffusion equation:

(10) $$\begin{array}{c} \frac{\partial p(x,t)}{\partial t}=-\sum_{i}\frac{\partial }{\partial x_{i}}\left[f_{i}\left(x,t\right)p\left(x,t\right)\right]+d\sum_{i}\sum_{j}\frac{\partial^{2}}{\partial x_{i}\partial x_{j}}p\left(x,t\right) \end{array}$$

where p(x,t) is the density function, and $f_{i}\left(x,t\right)$ (i=1,…,N) is the driving force of the system.

Given the system state described by p(x,t), we expect to have N‐dimensional partial differential equation, which are hard to solve for a high‐dimensional system. Here, we use the Gaussian distribution along the deterministic trajectory to approximate the time evolution of the density function of the system. For a Gaussian distribution, once we know two moments, the mean and the variance, we can obtain the probability distribution. Therefore, we only need to calculate the mean and the variance. When the diffusion coefficient d is small, the moment equations satisfy the following equations^[^ ^35^, ^36^ ^]^:

(11) $$\dot{\bar{x}}\left(t\right)=f\left(\bar{x}\left(t\right)\right)$$

(12) $$\dot{\Sigma }\left(t\right)=\Sigma \left(t\right)A^{T}\left(t\right)+A\left(t\right)\Sigma \left(t\right)+2d\cdot I$$

Here, Σ(t) denotes the covariance matrix in time t, I is identity matrix, and A(t) is the jacobian matrix of f(x) when x is equal to the solution of the deterministic equation $\bar{x}\left(t\right)$, which means $A_{ij}\left(t\right)=\frac{\partial f_{i}\left(x\right)}{\partial x_{j}}{|}_{x=\bar{x}\left(t\right)}$. Then the time evolution of the density function p(x,t) for this system can be expressed by:

(13) $$p\left(x,t\right)=\frac{1}{{\left(2\pi \right)}^{\frac{N}{2}}{\left|\Sigma \left(t\right)\right|}^{1/2}}\exp\left\{-\frac{1}{2}{\left(x-\bar{x}\left(t\right)\right)}^{T}\Sigma^{-1}\left(t\right)\left(x-\bar{x}\left(t\right)\right)\right\}$$

When t→+∞, we can get the density function p(x) of the system at the steady state. If the deterministic equation has multiple stable states, we use weighted sum of Gaussian distributions to represent the probability density function of the system:

(14) $$p\left(x\right)=\sum_{j=1}^{M}\phi^{j}p^{j}\left(x\right)$$

where $p^{j}\left(\cdot \right)$ is the density function corresponding to the jth stable state of the system, and $\phi_{j}$ (j=1,…,M) denotes corresponding weight. The weight $\phi^{j}$ represents the relative size of different basins of attraction. We determine the weights by giving a large number of random initial conditions to the ODEs to be solved, and then the frequency of each stable state is calculated as the weight of the corresponding stable state.

### 4.2 Dimension Reduction of Landscape (DRL)

To preserve as much information of the original landscape as possible after dimension reduction, we chose directions that contain the larger amount of information, that is, the directions with the larger variance. For example, we can project the random variable to a new coordinate system such that the largest variance of the random variable comes to lie on the first coordinate, the second largest variance on the second coordinate, and so on. Based on the idea of PCA, the N‐dimensional density function p(x) obtained from the TME method can be reduced to C dimensions (C<N) via an orthogonal linear transformation, and this C directions correspond to the directions with the top C largest variance of p(x).

The density function of the random variable X from the TME method^[^ ^19^ ^]^ is denoted by:

(15) $$p\left(x\right)=\sum_{j=1}^{M}\phi^{j}p^{j}\left(x\right)$$

where $p^{j}\left(x\right)$ (j=1,…,M and M is the total number of stable steady states) represents the density function of jth stable state, $\phi^{j}$ is the weight for corresponding stable state, and $X={\left(X_{1},X_{2},\ldots ,X_{N}\right)}^{T}$ is the random variable with N denoting the total number of variables. For any given stable state j, the random variable $X^{j}={\left(X_{1}^{j},X_{2}^{j},\ldots ,X_{N}^{j}\right)}^{T}$ follows a multi‐dimensional normal distribution with expectation vector $\mu_{j}$ and invertible covariance matrix $\Sigma_{j}$. That is, the density function of $X^{j}$ is given by

(16) $$p^{j}\left(x^{j}\right)=\frac{1}{{\left(2\pi \right)}^{\frac{N}{2}}{\left|\Sigma^{j}\right|}^{\frac{1}{2}}}\exp\left\{-\frac{1}{2}{\left(x^{j}-\mu^{j}\right)}^{T}{\left(\Sigma^{j}\right)}^{-1}\left(x^{j}-\mu^{j}\right)\right\}$$

We know that the expectation and covariance of the random variable X with multistability are given by: $\mu =\sum_{j=1}^{M}\phi^{j}\mu^{j}$, $\Sigma =\sum_{j=1}^{M}\phi^{j}\left(\Sigma^{j}+\mu^{j}{\left(\mu^{j}\right)}^{T}\right)-\mu \mu^{T}$ (see Methods in Supporting Information for details). Next, in order to choose the directions with the largest variance, we do an orthogonal linear transformation to the random variable X such that the largest variance of X falls on the first coordinate, the second largest variance falls on the second coordinate, and so on. In particular, we do the eigenvalue decomposition of the covariance matrix Σ and sort the obtained eigenvalues: $\lambda_{1}\geq \lambda_{2}\geq \ldots \geq \lambda_{N}$. Then, the eigenvectors corresponding to the eigenvalue sequentially form a matrix $W=(w_{1},\ldots ,w_{N})$, $w_{i}w_{j}^{T}=0,\forall i\neq j$, $∥w_{i}∥=1$, which is the new coordinates after transformation.

Let $Z^{j}=W^{T}X^{j}={\left(Z_{1}^{j},\ldots ,Z_{N}^{j}\right)}^{T},j=1,\ldots ,M$. Obviously, $Z^{j}$ also follows a multi‐dimensional normal distribution with expectation vector $\mu_{z}^{j}=W^{T}\mu^{j}$ and covariance matrix $\Sigma_{z}^{j}=W^{T}\Sigma^{j}W$, namely,

(17) $$\begin{array}{ccc} p_{z}^{j}\left(z^{j}\right) & = & p^{j}\left(Wz^{j}\right)\left|W\right|\\ & = & \frac{1}{{\left(2\pi \right)}^{\frac{N}{2}}{\left|\Sigma_{z}^{j}\right|}^{\frac{1}{2}}}\exp\left\{-\frac{1}{2}{\left(z^{j}-\mu_{z}^{j}\right)}^{T}{\left(\Sigma_{z}^{j}\right)}^{-1}\left(z^{j}-\mu_{z}^{j}\right)\right\} \end{array}$$

In a similar fashion, let $Z=W^{T}X={\left(Z_{1},\ldots ,Z_{N}\right)}^{T}$, and the density function $p_{z}$ of Z can be computed as

(18) $$\begin{array}{ccc} p_{z}\left(z\right) & = & p\left(Wz\right)|W|=\sum_{j=1}^{M}\phi_{j}p_{j}\left(Wz\right)=\sum_{j=1}^{M}\phi_{j}p_{z_{j}}\left(z\right) \end{array}$$

Now, let $W_{C}=\left(w_{1},\ldots ,w_{C}\right)$, $Z_{C}=W_{C}^{T}X$ and $Z_{C}^{j}=W_{C}^{T}X^{j}$, that is, $Z_{C}={\left(Z_{1},\ldots ,Z_{C}\right)}^{T}$, $Z_{C}^{j}={\left(Z_{1}^{j},\ldots ,Z_{C}^{j}\right)}^{T}$, where C is the dimensionality after dimension reduction (C≤N). Then, the density function of $Z_{C}$ is given by

(19) $$\begin{array}{ccc} p_{z_{C}}\left(z_{C}\right) & = & \int p_{z}\left(z\right)dz_{C+1}\cdots dz_{N}\\ & = & \int \sum_{j=1}^{M}\phi^{j}p_{z}^{j}\left(z\right)dz_{C+1}\cdots dz_{N}\\ & = & \sum_{j=1}^{M}\phi^{j}\int p_{z}^{j}\left(z\right)dz_{C+1}\cdots dz_{N}\\ & = & \sum_{j=1}^{M}\phi^{j}p_{z_{C}}^{j}\left(z_{C}\right), \end{array}$$

where $p_{z_{C}}^{j}\left(\cdot \right)$ (j=1,…,M) is a multi‐dimensional normal distribution, with the expectation vector being the vector composed of the first C components of $\mu_{z}^{j}$ and the covariance matrix being the matrix composed of the first C×C components of $\Sigma_{z}^{j}$.

In this way, we project the random variable X into the lower‐dimensional space constituted by $\left(w_{1},\ldots ,w_{C}\right)$. When C=2, the 2D landscape after dimension reduction can be calculated by $U_{2}=-\ln p_{z_{2}}\left(z_{2}\right)$.^[^ ^7^, ^8^, ^10^ ^]^

### 4.3 Minimum Action Paths (MAPs)

Considering a stochastic differential equation:

(20) $$dx\left(t\right)=f\left(x\right)dt+\sqrt{\varepsilon }dW\left(t\right)$$

where f(x) does not explicitly depend on the independent variable t, and W(t) is a Wiener process. Assuming that the initial time is 0 and the terminal time is T, we define the path between the ith attractor $x^{i}$ and jth attractor $x^{j}$ as $x^{ij}\left(t\right)={\left(x_{1}^{ij}\left(t\right),x_{2}^{ij}\left(t\right),\ldots ,x_{N}^{ij}\left(t\right)\right)}^{T}$ for t∈[0,T], where the path $x^{ij}\left(t\right)$ should satisfy the following boundary conditions:

(21) $$\left\{\begin{array}{c} {\left(x_{1}^{ij}\left(0\right),x_{2}^{ij}\left(0\right),\ldots ,x_{N}^{ij}\left(0\right)\right)}^{T}=x^{i}\\ {\left(x_{1}^{ij}\left(T\right),x_{2}^{ij}\left(T\right),\ldots ,x_{N}^{ij}\left(T\right)\right)}^{T}=x^{j} \end{array}\right.$$

The transition action $S^{ij}$ between $x^{i}$ and $x^{j}$ is defined as:

(22) $$\begin{array}{c} S^{ij}\left(x^{ij}\left(\cdot \right)\right)=\frac{1}{2}\int_{0}^{T}{\left|\left|{\left(\frac{dx_{1}^{ij}\left(t\right)}{dt},\frac{dx_{2}^{ij}\left(t\right)}{dt},\ldots ,\frac{dx_{N}^{ij}\left(t\right)}{dt}\right)}^{T}-f\left(x^{ij}\left(t\right)\right)\right|\right|}_{2}dt \end{array}$$

For a given δ, the Wentzell–Freidlin theory^[^ ^61^ ^]^ gives an estimate of the probability distribution of the solution x(t) over any fixed time interval [0,T]:

(23) $$P\left\{\rho \left(x\left(t\right),x^{ij}\left(t\right)\right)<\delta \right\}\approx exp\left(-\frac{S_{ij}\left(x^{ij}\right)}{\varepsilon }\right)$$

Following the approaches,^[^ ^46^, ^47^ ^]^ the most probable transition path connecting the ith attractor and jth attractor over the time interval [0,T] can be acquired through minimizing the transition action functional over all possible paths $\min_{x^{ij}\left(\cdot \right)}S^{ij}\left(x^{ij}\left(\cdot \right)\right)$. We calculated MAPs numerically by applying minimum action methods used in ref. [47], and treated the MAPs as the biological paths in our models.

## Conflict of Interest

The authors declare no conflict of interest.

## Supporting information

Supporting Information

## Data Availability

A matlab implementation of DRL approach has been deposited at GitHub (https://github.com/chunhelilab/DRL).
