# Supplementary material for: A Dimension Reduction Approach for Energy Landscape: Identifying Intermediate States in Metabolism‐EMT Network
Source: Adv Sci (Weinh). 2021 Mar 18;8(10):2003133. doi: 10.1002/advs.202003133 (PMC8132071; doi:10.1002/advs.202003133)
Supplement: Supplementary file 1 — Supporting Information [file ADVS-8-2003133-s001.pdf]

© 2021 Wiley-VCH GmbH

ADVANCED SCIENCE
Open Access

Supporting Information

for *Adv. Sci.*, DOI: 10.1002/advs.202003133

A Dimension Reduction Approach for Energy Landscape: Identifying Intermediate States in Metabolism-EMT Network

Xin Kang[1,2] *and Chunhe Li*[2,3,*]

Supporting Information

A Dimension Reduction Approach for Energy Landscape: Identifying Intermediate States in Metabolism-EMT Network

Xin Kang[1,2], Chunhe Li[2,3,*]

1 School of Mathematical Sciences, Fudan University, Shanghai, China
2 Shanghai Center for Mathematical Sciences, Fudan University, Shanghai, China
3 Institute of Science and Technology for Brain-Inspired Intelligence, Fudan University, Shanghai, China
∗ E-mail: chunheli@fudan.edu.cn

Supplemental Methods

Lemma

The density function of the random variable $\mathbf{X}$ from the TME method [1] is denoted by:

$$p(\mathbf{x}) = \sum_{j=1}^{M} \phi^j p^j(\mathbf{x}), \tag{S1}$$

where $p^j(x)$ ($j = 1, \cdots, M$ and M is the total number of stable steady states) represents the density function of jth stable state, ϕ^j is the weight for corresponding stable state, and $\mathbf{X} = (X_1, X_2, \cdots, X_N)^T$ is the random variable with N denoting the total number of variables. Then the expectation and covariance of the random variable $\mathbf{X}$ can be calculated by the following Lemma.

Lemma 1: When the density function of the random variable $\mathbf{X} = (X_1, X_2, \cdots, X_N)$ is denoted by:

$$p(\mathbf{x}) = \sum_{j=1}^{M} \phi^j p^j(\mathbf{x}), \tag{S2}$$

the expectation and covariance of the random variable $\mathbf{X}$ are given by

$$\mu = \sum_{j=1}^{M} \phi^j \mu^j, \tag{S3}$$

$$\Sigma = \sum_{j=1}^{M} \phi^j (\Sigma^j + \mu^j (\mu^j)^T) - \mu\mu^T, \tag{S4}$$

where μ^j and Σ^j are the expectation vector and invertible covariance matrix of $\mathbf{X}^j$ (the the density function of $\mathbf{X}^j$ is $p^j(\mathbf{x})$).

Proof: For any $1 \le i \le N$,

$$\begin{aligned}
\mathrm{E}(X_i) &= \int x_i p(\mathbf{x}) dx_1 \cdots dx_N \\
&= \int x_i \sum_{j=1}^{M} \phi^j p^j(\mathbf{x}) dx_1 \cdots dx_N \\
&= \sum_{j=1}^{M} \phi^j \int x_i p^j(\mathbf{x}) dx_1 \cdots dx_N \\
&= \sum_{j=1}^{M} \phi^j \mathrm{E}(X_i^j).
\end{aligned}$$

Therefore, the expectation of $\mathbf{X}$ can be computed as

$$\mu = \mathrm{E}(\mathbf{X}) = \sum_{j=1}^{M} \phi^j \mathrm{E}(\mathbf{X}^j) = \sum_{j=1}^{M} \phi^j \mu^j.$$

Also, for any $1 \le p, q \le N$,

$$\begin{aligned}
\mathrm{Cov}(X_p, X_q) &= \int (x_p - \mathrm{E}(X_p))(x_q - \mathrm{E}(X_q)) p(x) dx_1 \cdots dx_N \\
&= \int (x_p - \mathrm{E}(X_p))(x_q - \mathrm{E}(X_q)) \sum_{j=1}^{M} \phi^j p^j(x) dx_1 \cdots dx_N \\
&= \sum_{j=1}^{M} \phi^j \int (x_p - \mathrm{E}(X_p))(x_q - \mathrm{E}(X_q)) p^j(x) dx_1 \cdots dx_N \\
&= \sum_{j=1}^{M} \phi^j \int (x_p x_q - x_p \mathrm{E}(X_q) - x_q \mathrm{E}(X_p) + \mathrm{E}(X_p)\mathrm{E}(X_q)) p^j(x) dx_1 \cdots dx_N \\
&= \sum_{j=1}^{M} \phi^j \left(\mathrm{E}(X_p^j X_q^j) - \mathrm{E}(X_p^j)\mathrm{E}(X_q) - \mathrm{E}(X_q^j)\mathrm{E}(X_p) + \mathrm{E}(X_p)\mathrm{E}(X_q) \right) \\
&= \sum_{j=1}^{M} \phi^j \left(\mathrm{Cov}(X_p^j, X_q^j) + \mathrm{E}(X_p^j)\mathrm{E}(X_q^j) \right) - \mathrm{E}(X_p)\mathrm{E}(X_q).
\end{aligned}$$

Hence, the covariance matrix of $\mathbf{X}$ is given by

$$\begin{aligned}
\Sigma &= \mathrm{Cov}(\mathbf{X}) = \sum_{j=1}^{M} \phi^j \left(\mathrm{Cov}(\mathbf{X}^j, \mathbf{X}^j) + \mathrm{E}(\mathbf{X}^j)\mathrm{E}(\mathbf{X}^j)^T \right) - \mathrm{E}(\mathbf{X})\mathrm{E}(\mathbf{X})^T \\
&= \sum_{j=1}^{M} \phi^j \left(\Sigma^j + \mu^j (\mu^j)^T \right) - \mu\mu^T.
\end{aligned}$$

□

Simplified ODEs for Gene Regulatory Network

For simplicity, we have unified the parameters of MISA, MESC, and HESC model, we set all the activation parameters to a unified a, all the inhibition parameters to a unified b, and all the threshold parameters to a unified S. Then the

ODEs have the form as:

$$f_i(\mathbf{x}) = \frac{dx_i}{dt} = \sum_{j=1}^{N} \frac{a \times x_j^n}{S^n + x_j^n} + \sum_{j=1}^{N} \frac{b \times S^n}{S^n + x_j^n} - k \times x_i, \tag{S5}$$

where x_i $(i = 1, \cdots, N)$ represents the expression level of gene i, k represents the basal degradation rates of x_i and n is the Hill coefficient.

Synthetic Multistable Gene Regulatory Network

To see how the DRL approach works for a gene network including more stable states, we studied a synthetic multistable gene network (Figure S3A). In this model, as we increase the number of positive feedbacks, we can have increasing number of stable states. For example, as we use four nodes with self-activations, we obtained 7 stable states (Table S1, Figure S3B and C). Based on the network in Figure S3A, we constructed the ODEs. The parameters in this model are: $a = 0.35$, $b = 0.3$, $S = 0.2$, $n = 4$, $k = 1$. There are 7 stable points in the original space and the expression levels of the stable states are shown in Table S1. We further showed the landscape and paths of this system with 7 stable states in three-dimensional and two-dimensional landscape by our dimension reduction approach (Figure S3B and C). Figure S3B is the landscape, paths, and the stable points shown in PC1-PC2-PC3 coordinates. Figure S3C is the landscape, paths, and the stable points shown in PC1-PC2 coordinates.

After reducing the dimensionality to three, the stable points are still scattered in different positions. However, when the dimensionality is reduced to two, there will be two stable states that almost overlap each other, namely the stable state 5 and 6 in Figure S3C. This may be due to the fact that the number of stable states of this system is much greater than two, so the two-dimensional coordinate system may not carry sufficient information of this system, which can be seen from that the total contribution rate of PC1, PC2, and PC3 (94.52%) is greater than that of PC1 and PC2 (76.68%). We also showed the transition paths in the direction from state 1 to state 7 (cyan paths) and the transition paths in reverse direction (magenta paths), individually. For this network, we calculated the potential energy of each stable state in different dimensional spaces. From Figure S3D, we see that the potential energy of each stable state in different dimensional space has a similar trend as the parameter changes except the stable state 5 and 6, which indicates that both the three-dimensional landscape and two-dimensional landscape can retain the major information on the stability of each stable state in original system, but the three-dimensional landscape is more accurate than the two-dimensional one. We also changed the regulation strength a to investigate the correlation among the RBH in different dimensional landscape (Figure S3E). As an example, we chose stable state 1 and 4, as well as stable state 4 and 7 to calculate ΔS and RHB. From Figure S3E, we see that the relative stability of stable states in two-dimensional landscape, three-dimensional landscape and in four-dimensional landscape are consistent. These results suggest that the landscape after dimension reduction preserves the information of relative stability and transition of this multistable system, and the DRL approach has the potential to be applied to the system with many stable states.

Parameter Search Algorithm for metabolism-EMT model

Recently, it has been proposed that multiple intermediate states exist during EMT [2]. Many studies have shown that there are an intermediate state H (hybrid E/M cell state) of EMT [3–7], and our previous work identified an intermediate abnormal metabolic cell state (A) [1]. Here, we used a random parameter search strategy to search the parameter sets where H state and A state can coexist. That is, we chose the parameter sets that can generate quadrastable states (E, A, H, and M state). We started from a random parameter set p_0 (the initial parameters are shown in Table S11), and repeated following steps 10000 times:

1. We solved the ODEs under the current parameter set.

2. If there are four stable states (M state has higher expression level of HIF-1 and ZEB1, lower expression level of miR-145, miR-200 and miR-34; E state has lower expression level of HIF-1, ZEB1, higher expression level of miR-145, miR-200 and miR-34; A state has lower expression level of HIF-1, higher expression level of ZEB1 and

H state has middle expression levels of HIF-1 and ZEB1) under the current parameter set, accept the current parameter set.

3. Now at parameter set p,

   - if there are four stable states under p, propose a move to p' according to $p' \sim N(p, 0.1I)$, that is, give a small perturbation to parameter set p. Here, the small perturbation means that since we already reach the parameter region for four stable states we don't want to go too far from four stable states.
   - otherwise, propose a move to p' according to $p' \sim N(p, 0.5I)$, that is, give a large perturbation to parameter set p. Here, the large perturbation means that since we have not found the parameter region for four stable states we need a larger perturbations for parameter set.

where I is an identity matrix. Then, we picked one of the parameter sets which has E, M, A and H states as the default parameters of the model (the default parameters are shown in Table S7).

Supplemental Figures

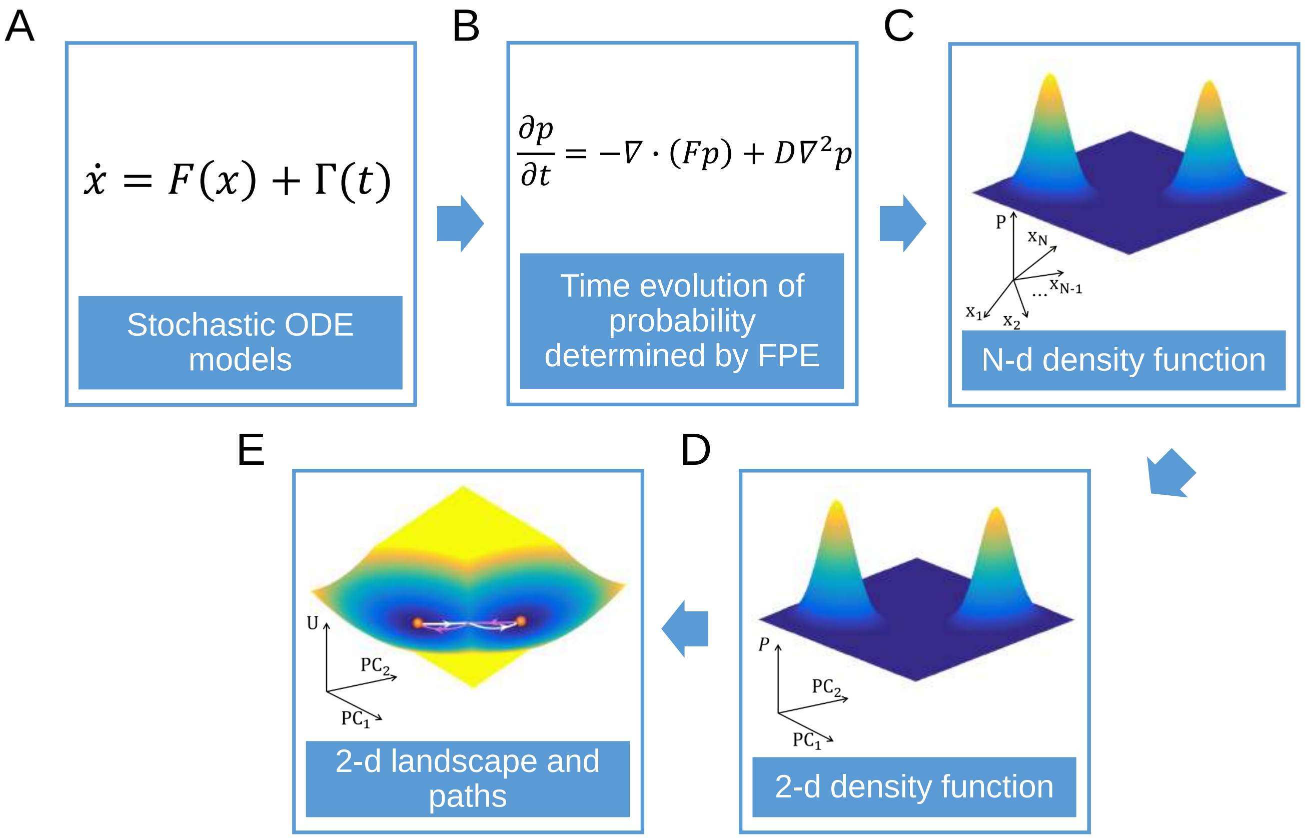

Figure S1: **The overview of the scheme for the DRL approach.** (A) The ordinary differential equations (ODEs) are constructed according to the network topology. (B) The time evolution of the dynamical system is determined by probabilistic diffusion equations (Fokker-Planck equation). (C) The solution of the FPE can be calculated from Gaussian approximation. (D) The density function can be projected in a two-dimensional space by the DRL approach. (E) The potential landscape is calculated by $U(x) = lnP_{ss}(x)$.

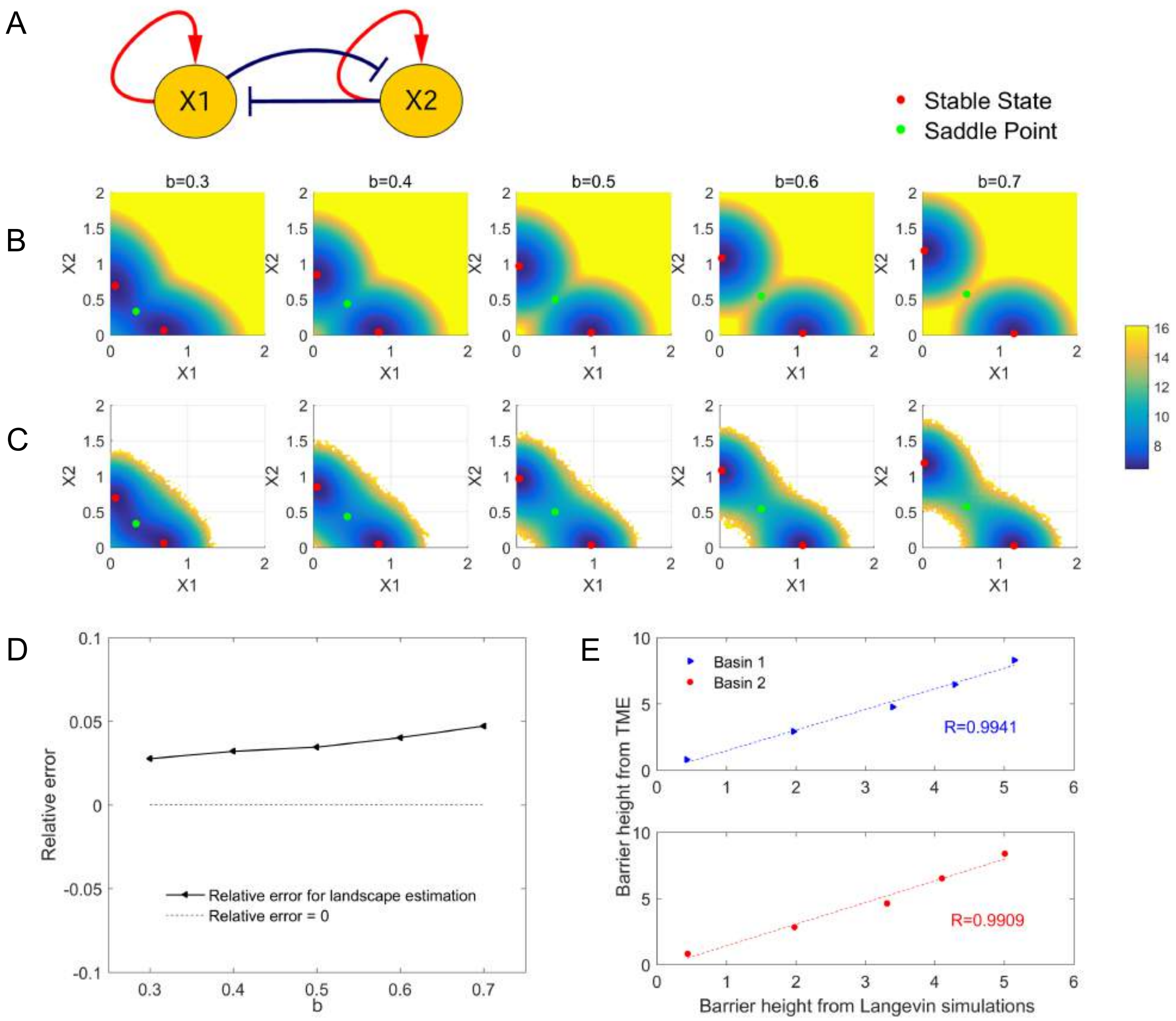

Figure S2: **The comparison between the TME method and Langevin simulations for MISA model.** (A) The network of the MISA model including 2 nodes. (B) Landscape calculated by TME method at different parameter b (inhibition constant). (C) Landscape calculated by Langevin simulations at different parameter b (inhibition constant). (D) The relative error for the landscape estimation by TME method compared with Langevin simulations. (E) The correlation for the potential barrier height of the landscape calculated by the TME method and that by Langevin simulations. The correlation coefficients are 0.9941 and 0.9909, for two basins, respectively.

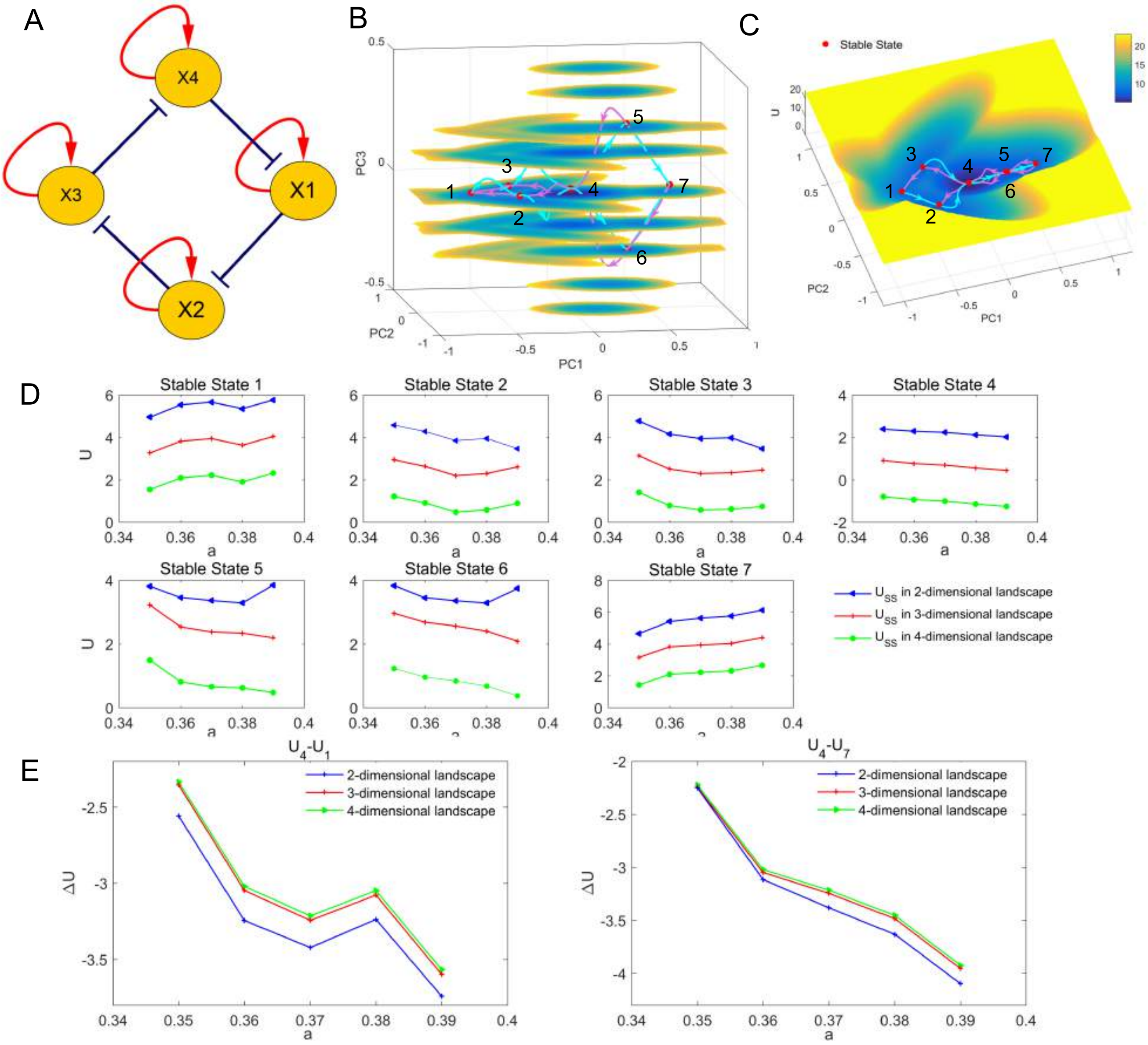

Figure S3: **The dimension reduction of synthetic multistable gene regulatory model.** (A) Multistable gene regulatory network. (B) Landscape and paths of multistable gene regulatory network shown in PC1-PC2-PC3 coordinates. The total contribution rate of PC1, PC2 and PC3 is 94.52%. The cyan and magenta paths represent the transition paths in the direction from state 1 to state 7 and the transition paths in reverse direction, individually. (C) Landscape and paths of multistable gene regulatory network shown in PC1 and PC2 coordinates. The total contribution rate of PC1 and PC2 is 76.68%. The stable point 5 and stable point 6 in (B) correspond to one point in (C). The expression levels of the stable states are shown in Table S1. (D) The potential energy of each stable state changes with parameter a (self-activation constant) in two, three, and four-dimensional space. (E) The relative barrier height changes with a in two, three, and four-dimensional landscape.

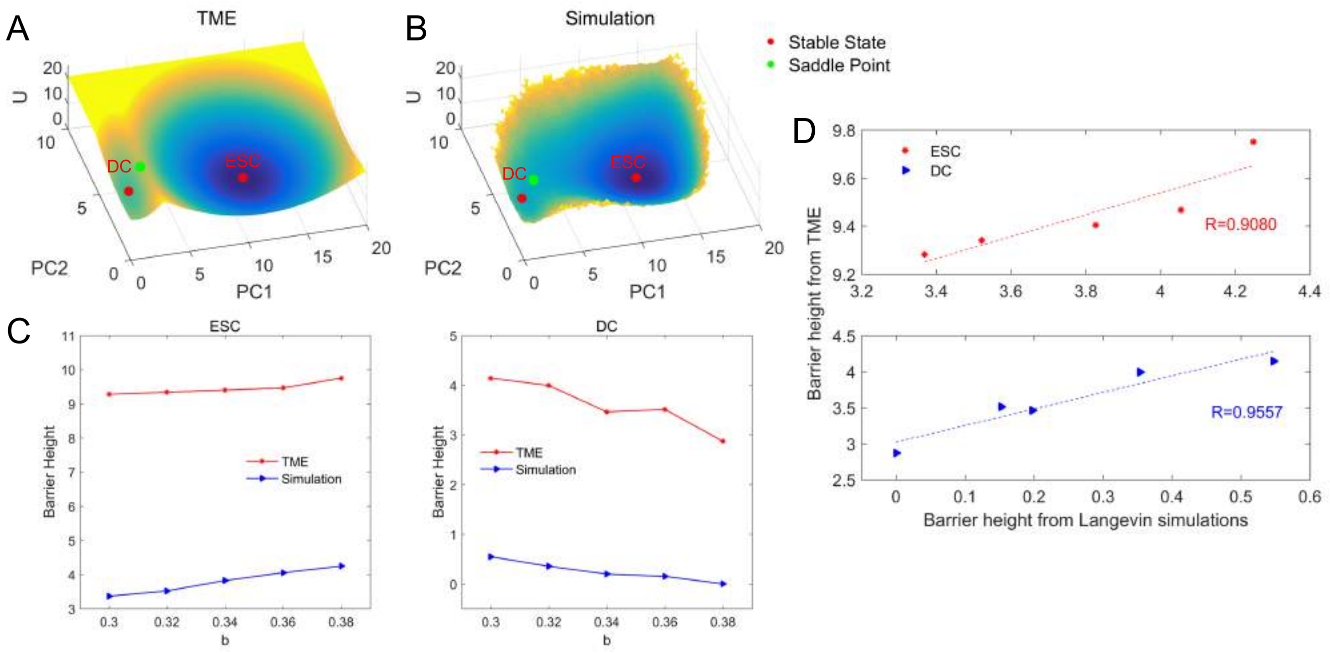

Figure S4: **The comparison between TME method and Langevin simulations for the MESC model.** (A) Landscape of MESC network calculated by TME and DRL method (projected on PC1 and PC2 space). (B) Landscape of MESC network calculated by Langevin simulations. (C) The barrier height (the potential difference between a saddle point and a stable point) of each basin (ESC basin and DC basin) changes with parameter b (inhibition constant). (D) The correlation between the barrier height calculated by TME and that by Langevin simulations. The correlation coefficients are 0.9080 and 0.9557, for two basins, respectively. ESC: embryonic stem cell state; DC: differentiated cell state.

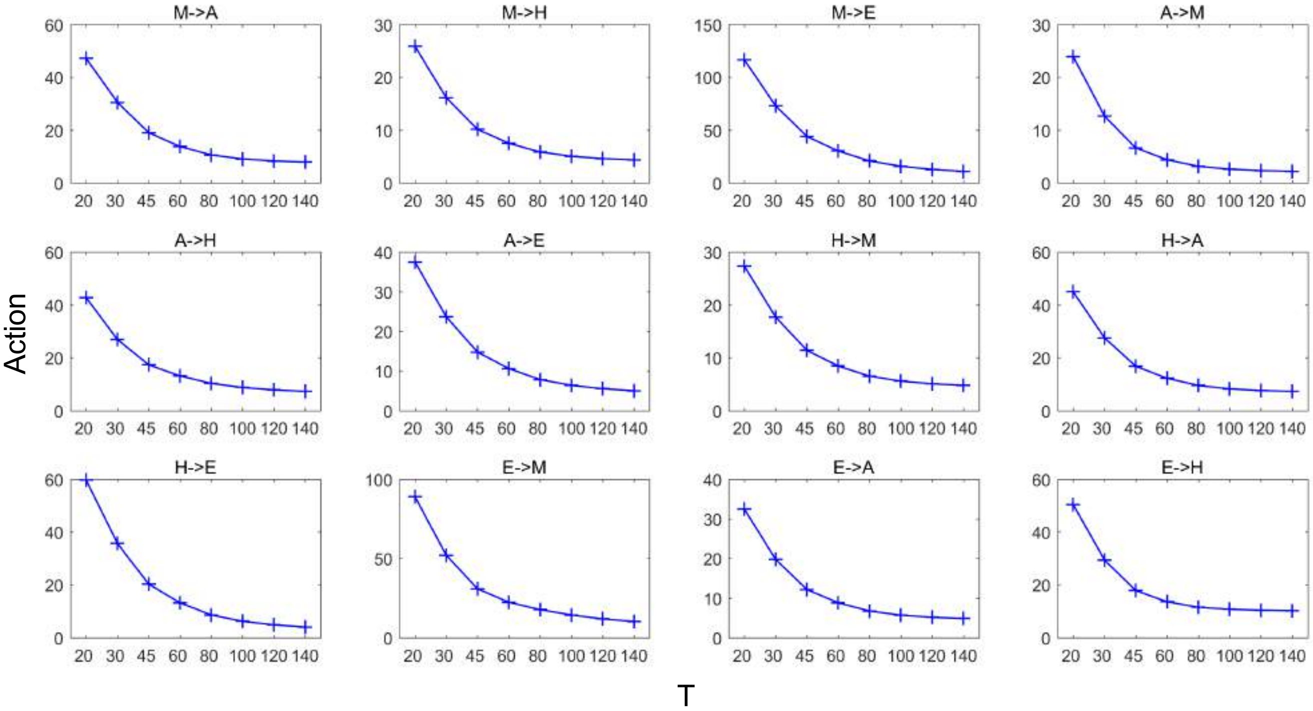

Figure S5: **The transition actions between two stable states change with terminal time T for the metabolism-EMT model.** The transition action converges as the termination time goes on, and when the termination time $T > 120$, all the transition actions tend to level off. E: epithelial cell state, A: abnormal metabolic cell state, H: hybrid E/M cell state, M: mesenchymal cell state.

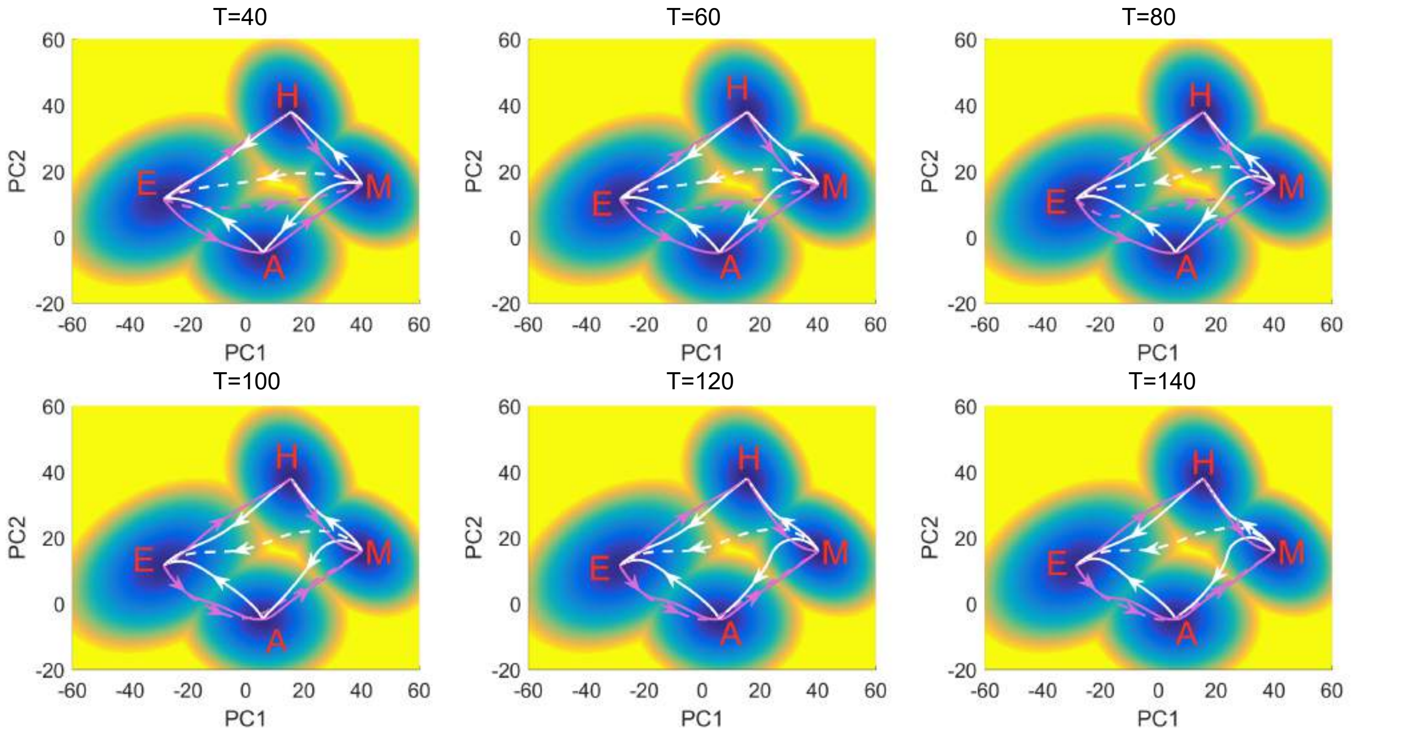

Figure S6: **The transition paths of the metabolism-EMT model for different terminal time** T **(**$T = 40, 60, \cdots, 140$**).** The transition paths have a similar pattern when $T \geq 100$. E: epithelial cell state, A: abnormal metabolic cell state, H: hybrid E/M cell state, M: mesenchymal cell state.

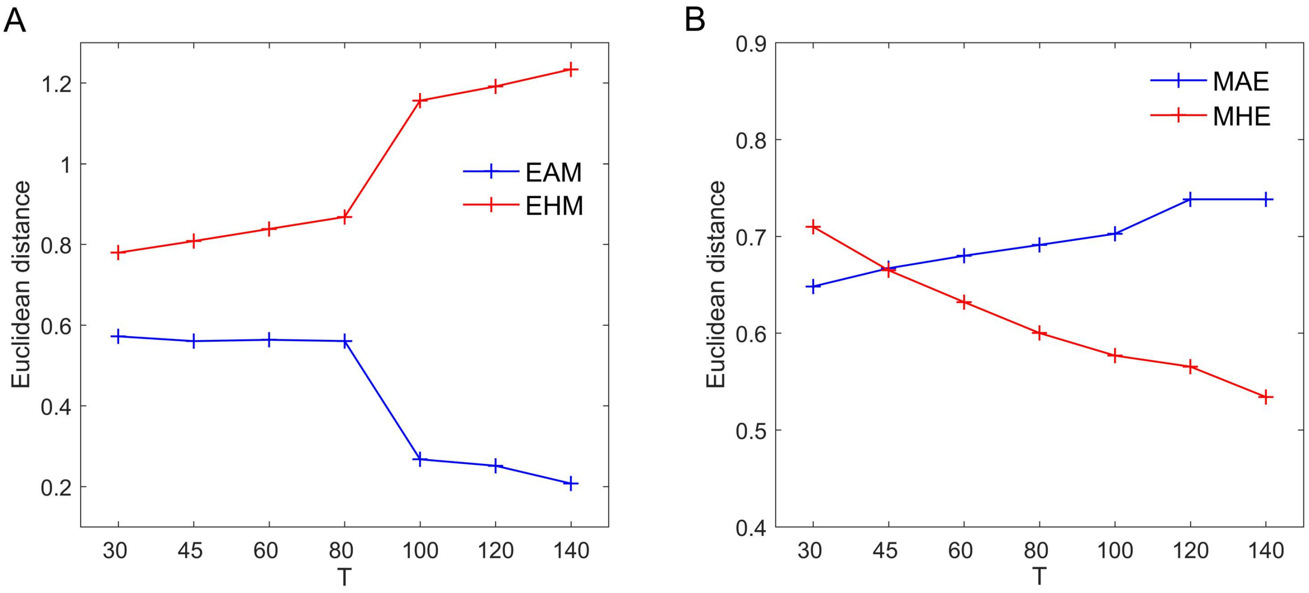

Figure S7: **The Euclidean distance between the indirect transition paths and the direct transition paths for the metabolism-EMT model.** (A) The Euclidean distance between the indirect transition paths from E state to M state and the direct transition paths from E state to M state. (B) The Euclidean distance between the indirect transition paths from M state to E state and the direct transition paths from M state to E state. Y axis represents the Euclidean distance. X axis represents the different terminal time T. When $T > 100$, the transition path from E state to M state tends to first go through the A state and the transition path from M state to E state is more likely to first go through the H state. E: epithelial cell state, A: abnormal metabolic cell state, H: hybrid E/M cell state, M: mesenchymal cell state.

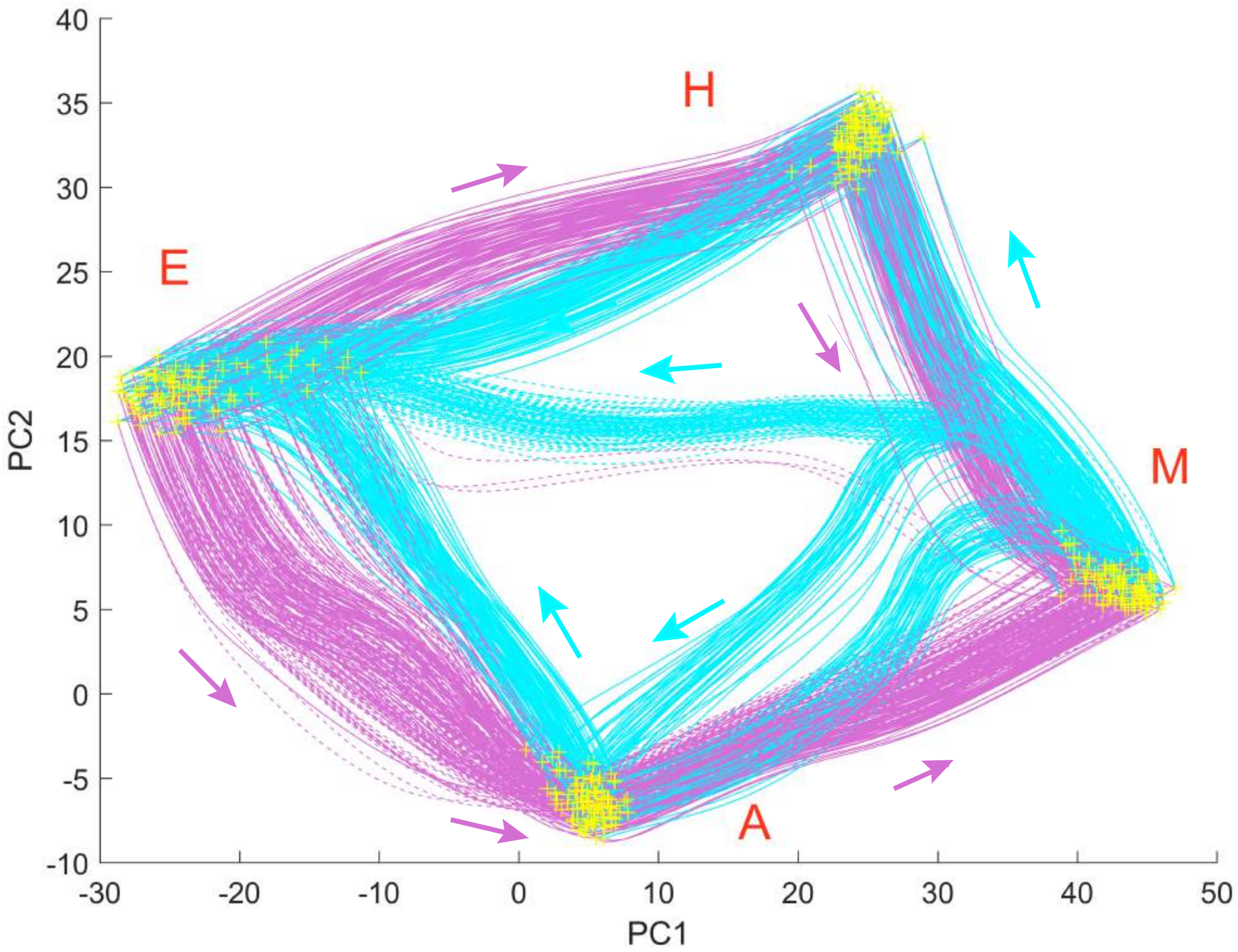

Figure S8: **The stable states and transition paths from 100 parameter sets shown in PC1 and PC2 coordinates for the metabolism-EMT model.** Yellow points denote the stable states. The magenta paths denote the transitions from E state to M state, while the cyan paths denote the transitions from M state to E state. The solid links represent the indirect paths between E state and M state, and the dashed links represent direct paths between E state and M state. E: epithelial cell state, A: abnormal metabolic cell state, H: hybrid E/M cell state, M: mesenchymal cell state.

Supplemental Tables

Table S1: **The gene expression levels of the stable states for the synthetic multistable gene regulatory network (7 stable states)**.

	1	2	3	4	5	6	7
X1	0.003	0.020	0.280	0.345	0.387	0.647	0.647
X2	0.647	0.647	0.387	0.345	0.020	0.282	0.003
X3	0.003	0.282	0.020	0.345	0.647	0.387	0.647
X4	0.647	0.387	0.647	0.345	0.282	0.020	0.003

Table S2: **Connections for the MESC network [8]**. For the column of interaction type, 1 represents activation, and -1 represents inhibition.

Source Node	Target Node	Interaction Type
LIF	Stat3	1
CH	Tcf3	-1
PD	MEKERK	-1
Stat3	Gbx2	1
Stat3	Klf4	1
Klf4	Klf2	1
Stat3	Tlfcp2l1	1
Klf4	Tlfcp2l1	1
Sall4	Klf2	1
Tlfcp2l1	Sall4	1
Tcf3	Tlfcp2l1	-1
Tcf3	Esrrb	-1
Tlfcp2l1	Esrrb	1
Esrrb	Tlfcp2l1	1
Sall4	Sox2	1
Klf2	OCT4	1
Sox2	OCT4	1
OCT4	Tlfcp2l1	-1
OCT4	Nanog	1
Klf2	Nanog	1
Esrrb	OCT4	-1
Nanog	Esrrb	1
MEKERK	Nanog	-1
MEKERK	Tcf3	-1
Nanog	Sox2	1
Gbx2	Klf4	1

Table S3: **The new coordinates after dimension reduction for MESC network.** The coordinates of a point in the PC1-PC2 plane can be represented by $(z_1, z_2)^T = (w_1^T \mathbf{x}, w_2^T \mathbf{x})^T$, where w_1 and w_2 are the top two principal components and $\mathbf{x}$ is the coordinate of the corresponding point in the original high-dimensional space. The contribution rate equals to the ratio of the ith eigenvalue to the sum of all eigenvalues, which represents the proportion of the original information carried by the current principal component.

	Gene	w_1	w_2
1	Mekerk	0	0
2	OCT4	0.38	0.29
3	Sox2	0.49	0.03
4	Nanog	0.4	0.35
5	Esrrb	0.53	-0.21
6	Klf2	0.21	0.12
7	Tfcp2l1	0.27	-0.54
8	Klf4	0	-0.43
9	Gbx2	0	-0.40
10	Tcf3	0	0.00
11	Sall4	0.25	-0.12
12	Stat3	0	-0.29
contribution rate		95.52%	1.15%

Table S4: **Connections for the HESC network [9]**. For the column of interaction type, 1 represents activation, and -1 represents inhibition.

Source Node	Target Node	Interaction Type	Source Node	Target Node	Interaction Type
OCT4	OCT4SOX2	1	PBX1	NANOG	1
OCT4	KLF4	1	FOXA2	FOXA2	1
OCT4	FOXD3	1	FOXA2	AFP	1
OCT4	ZFP42	1	SOX17	FOXA2	1
OCT4	TDGF1	1	GATA4	GATA6	-1
OCT4	SOX17	-1	GATA6	NANOG	-1
OCT4	GATA4	-1	GATA6	GATA6	1
OCT4	GATA6	-1	GATA2	hCGa	1
OCT4	T	-1	GATA3	hCGa	1
OCT4	hCGa	-1	CDX2	OCT4	-1
OCT4	hCGb	-1	CDX2	CDX2	1
OCT4	CDX2	-1	Bcat	LEF1bCat	1
OCT4	FOXO1A	1	BMP2K	BMP2	1
OCT4	LEF1	-1	Camp	hCGa	1
OCT4	OCT4FOXD3	1	Camp	hCGb	1
OCT4	PRDM14	1	Camp	LMCD1	-1
SOX2	OCT4SOX2	1	CEBP	SP1	1
SOX2	KLF4	1	E2F4	FOXD3	1
SOX2	FOXD3	1	E2F4	T	-1
SOX2	ZIC3	1	E2F4	CEBP	-1
SOX2	TDGF1	1	E2F4	MYC	1
SOX2	GATA2	-1	E2F4	NFYA	1
SOX2	BMP2	-1	E2F4	PIAS1	1
SOX2	FOXO1A	1	E2F4	SP1	1
SOX2	NFYA	1	E2F4	SUMO1	1
SOX2	PRDM14	1	E2F4	TCF3	1
SOX2	SALL4	1	FOXA1	AFP	1
NANOG	OCT4	1	FOXA1	FOXA1	1
NANOG	SOX2	1	LEF1	LEF1bCat	1
NANOG	NANOG	1	LEF1	SALL4	1
NANOG	KLF4	1	LEF1bCat	MYC	1
NANOG	FOXD3	1	LMCD1	GATA6	-1
NANOG	ZIC3	1	Mad	MadMax	1
NANOG	ZFP42	1	MAX	MadMax	1
NANOG	GDF3	1	MAX	MycMax	1
NANOG	TDGF1	1	MYC	MycMax	1
NANOG	PBX1	1	MYC	MYCSP1	1
NANOG	GATA4	-1	MYCSP1	SP1	-1
NANOG	GATA6	-1	NFYA	SP1	1
NANOG	T	-1	OCT4FOXD3	FOXA2	-1
NANOG	GATA2	-1	OCT4FOXD3	FOXA1	-1
NANOG	GATA3	-1	PIAS1	GATA4	1
NANOG	CDX2	-1	PIAS1	PIAS1	1
NANOG	BMP2	-1	PIAS1	PIASy	1
NANOG	FOXO1A	1	PIAS1	SP3	-1

NANOG	LEF1	-1	PIASy	GATA2	-1
NANOG	LMCD1	1	PRDM14	GATA6	-1
NANOG	PRDM14	1	PRDM14	T	-1
NANOG	SALL4	1	PRDM14	CSH1	-1
NANOG	SUMO1	1	PRDM14	MYC	1
NANOG	ZEF206	1	SP1	hCGb	1
OCT4SOX2	OCT4	1	SP1	CEBP	-1
OCT4SOX2	SOX2	1	SP1	MYCSP1	1
OCT4SOX2	NANOG	1	SP1	SP1	1
KLF4	NANOG	1	SP3	hCGb	1
FOXD3	FOXA2	1	SP3	SP1	-1
FOXD3	FOXA1	1	SUMO1	GATA4	1
FOXD3	OCT4FOXD3	1	SUMO1	GATA2	-1
ZIC3	NANOG	1	SUMO1	SP3	-1
ZIC3	SOX17	-1	TCF3	MYC	-1

Table S5: **The new coordinates after dimension reduction for HESC network.** The coordinates of a point in the PC1-PC2 plane can be represented by $(z_1, z_2)^T = (w_1^T \mathbf{x}, w_2^T \mathbf{x})^T$, where w_1 and w_2 are the top two principal components and $\mathbf{x}$ is the coordinate of the corresponding point in the original high-dimensional space. The contribution rate equals to the ratio of the ith eigenvalue to the sum of all eigenvalues, which represents the proportion of the original information carried by the current principal component.

	Gene	w_1	w_2
1	OCT4	0.24	-0.08
2	SOX2	0.23	0.27
3	NANOG	0.34	-0.01
4	OCT4SOX2	0.21	0.18
5	KLF4	0.23	0.04
6	FOXD3	0.23	0.04
7	ZIC3	0.19	0.09
8	ZFP42	0.11	-0.13
9	GDF3	0.06	-0.07
10	TDGF1	0.23	0.04
11	PBX1	0.07	-0.07
12	FOXA2	-0.07	0.18
13	AFP	-0.01	0.07
14	SOX17	-0.19	0.05
15	GATA4	-0.13	0.37
16	GATA6	-0.20	0.21
17	T	-0.23	0.25
18	GATA2	-0.26	-0.39
19	GATA3	-0.07	0.09
20	hCGa	-0.11	0.09
21	hCGb	-0.07	0.11
22	CDX2	-0.20	0.25
23	Bcat	0.00	0.00
24	BMP2	-0.23	-0.14
25	BMP2K	0.00	0.00

26	Camp	0.00	0.00
27	HNF4A	0.00	0.00
28	CEBP	-0.01	-0.03
29	CSH1	-0.08	0.07
30	E2F4	0.00	0.00
31	FOXA1	-0.01	0.10
32	FOXO1A	0.23	0.04
33	LEF1	-0.15	0.18
34	LEF1bCat	-0.05	0.07
35	LMCD1	0.06	-0.08
36	Mad	0.00	0.00
37	MadMax	0.00	0.00
38	MAX	0.00	0.00
39	MYC	0.05	-0.04
40	MycMax	0.02	-0.02
41	MYCSP1	0.02	-0.01
42	NFYA	0.11	0.19
43	OCT4FOXD3	0.11	-0.16
44	PIAS1	0.00	0.00
45	PIASy	0.00	0.00
46	PRDM14	0.23	0.02
47	SALL4	0.12	0.17
48	SP1	0.02	0.22
49	SP3	-0.03	-0.26
50	SUMO1	0.06	0.18
51	TCF3	0.00	0.00
52	ZEF206	0.06	-0.07
contribution rate		16.91%	4.17%

Table S6: **Connections for the Metabolism-EMT network [1]**. For the column of interaction type, 1 represents activation, and -1 represents inhibition.

Source Node	Target Node	Interaction Type	Source Node	Target Node	Interaction Type
noxROS	HIF-1	1	P53	miR-145	1
noxROS	AMPK	1	P53	miR-34	1
mtROS	HIF-1	1	MDM2	P53	-1
mtROS	AMPK	1	miR-145	MDM2	-1
AMPK	AMPK	-1	miR-145	OCT4	-1
AMPK	noxROS	-1	miR-145	ZEB1	-1
AMPK	mtROS	1	OCT4	miR-145	-1
AMPK	mtROS	-1	OCT4	OCT4	1
AMPK	P53	1	OCT4	miR-200	1
AMPK	HIF-1	-1	miR-200	ZEB1	-1
HIF-1	HIF-1	1	ZEB1	ZEB1	1
HIF-1	noxROS	1	ZEB1	miR-145	-1
HIF-1	mtROS	-1	ZEB1	miR-200	-1
HIF-1	AMPK	-1	ZEB1	miR-34	-1
HIF-1	ZEB1	1	SNAIL	ZEB1	1

HIF-1	P53	1	SNAIL	miR-34	-1
P53	HIF-1	-1	SNAIL	miR-200	-1
P53	miR-200	1	SNAIL	SNAIL	-1
P53	OCT4	-1	miR-34	SNAIL	-1
P53	MDM2	1			

Table S7: **Default parameters for the metabolism-EMT model**. The degradation rate k for each gene is set to 1 and the Hill coefficient for each gene is set to 4. Here, A and B are interaction matrix and S represents the threshold.

Interaction Type	A_{ji}/B_{ji}	S_{ji}	Interaction Type	A_{ji}/B_{ji}	S_{ji}
AMPK→mtROS	0.7	18	HIF⊣AMPK	2	13
HIF-1→noxROS	0.1	9	AMPK⊣AMPK	0.1	9
ROS→AMPK	1	19	AMPK⊣HIF-1	1.7	13
HIF-1→HIF-1	0.9	20	P53⊣HIF-1	0.3	5
ROS→HIF-1	0.8	17	miR200⊣ZEB1	0.9	14
SNIAL→ZEB1	1.5	12	miR145⊣ZEB1	1.8	15
HIF-1→ZEB1	0.6	11	P53⊣OCT4	0.7	15
ZEB1→ZEB1	1.2	19	miR145⊣OCT4	0.4	7
OCT4→OCT4	1.9	13	miR145⊣MDM2	1.8	14
P53→MDM2	0.7	11	SNIAL⊣SNAIL	1.8	14
P53→miR145	0.3	11	miR34⊣SNAIL	1.8	5
OCT4→miR200	0.1	6	OCT4⊣miR145	1.9	8
P53→miR200	0.8	17	ZEB1⊣miR145	1.9	16
P53→miR34	1.8	6	ZEB1⊣miR200	0.3	20
AMPK→P53	2	6	SNAIL⊣miR200	1.4	13
HIF-1→P53	0.9	17	SNAIL⊣miR34	0.5	10
AMPK⊣mrROS	0.7	11	ZEB1⊣miR34	0.2	15
HIF-1⊣mrROS	1.5	7	MDM2⊣P53	1	14
AMPK⊣noxROS	0.1	7			

Table S8: **The gene expression levels of the stable states for metabolism-EMT model.** E: Epithelial state, A: Abnormal metabolic state, H: hybrid E/M cell state, M: Mesenchymal state.

	M	A	H	E
mtROS	7.07	7.08	16.50	16.51
noxROS	1.94	1.94	0.12	0.12
AMPK	3.14	3.20	23.13	23.14
HIF-1	23.65	23.42	5.42	5.42
ZEB1	49.91	32.01	41.03	12.59
OCT4	27.92	2.55	22.21	0.45
MDM2	20.95	8.38	24.44	7.28
SNAIL	11.92	11.90	11.90	11.89
miR145	1.59	22.51	3.53	35.67
miR200	10.19	12.84	14.97	18.01
miR34	17.72	19.50	19.58	20.97
P53	10.16	17.40	20.97	29.32
PC1	42.67	4.92	23.37	-25.44
PC2	7.00	-6.08	33.59	17.45

Table S9: **The new coordinates after dimension reduction for metabolism-EMT network.** The coordinates of a point in the PC1-PC2 plane can be represented by $(z_1, z_2)^T = (w_1^T \mathbf{x}, w_2^T \mathbf{x})^T$, where w_1 and w_2 are the top two principal components and $\mathbf{x}$ is the coordinate of the corresponding point in the original high-dimensional space. The contribution rate equals to the ratio of the ith eigenvalue to the sum of all eigenvalues, which represents the proportion of the original information carried by the current principal component.

	Gene	w_1	w_2
1	mtROS	-0.1	0.28
2	noxROS	0.02	-0.05
3	AMPK	-0.2	0.60
4	HIF-1	0.19	-0.54
5	ZEB1	0.53	-0.01
6	OCT4	0.48	0.25
7	MDM2	0.24	0.29
8	SNAIL	0.00	0.00
9	miR145	-0.52	-0.25
10	miR200	-0.1	0.10
11	miR34	-0.05	0.03
12	P53	-0.25	0.21
contribution rate		71.36%	26.88%

Table S10: **Key links identified from global sensitivity analysis (Figure 7 in main text)**. Sensitivity is defined as the difference between $\triangle S_{M\to E}$ and $\triangle S_{E\to M}$ ($\triangle S_{M\to E} - \triangle S_{E\to M}$). Here, the inhibition constant B_{ji} represents the maximal synthesis rate when the inhibition regulation is completely not working. So, the increase of B_{ji} represents the decrease of the corresponding inhibition strength. A: The top 5 targets which can make M state more stable when each parameter is decreased by 10%; B: The top 5 targets which can make E state more stable when each parameter is decreased by 10%; C: The top 5 targets which can make E state more stable when each parameter is increased by 10%; D: The top 5 targets which can make M state more stable when each parameter is increased by 10%.

**A**		**B**	
Regulation	Sensitivity	Regulation	Sensitivity
HIF-1⊣ AMPK	0.1508	AMPK⊣HIF-1	-0.1783
AMPK→P53	0.0543	OCT4→OCT4	-0.1534
OCT4⊣miR145	0.0378	HIF-1→HIF-1	-0.0401
P53→miR145	0.0207	ZEB1→ZEB1	-0.0365
HIF-1→P53	0.0197	P53⊣OCT4	-0.0275
**C**		**D**	
Regulation	Sensitivity	Regulation	Sensitivity
HIF-1⊣AMPK	-0.1377	OCT4→OCT4	0.1666
SNAIL→ZEB1	-0.0491	AMPK⊣HIF-1	0.1633
SNAIL⊣SNAIL	-0.0468	HIF-1→HIF-1	0.0421
AMPK→P53	-0.0454	ZEB1→ZEB1	0.0308
OCT4⊣miR145	-0.0350	P53⊣OCT4	0.0267

Table S11: **Initial parameters of parameter search for the metabolism-EMT model**. The degradation rate k for each gene is set to 1 and the Hill coefficient for each gene is set to 4. Here, A and B are interaction matrix and S represents the threshold.

Interaction Type	A_{ji}/B_{ji}	S_{ji}	Interaction Type	A_{ji}/B_{ji}	S_{ji}
AMPK→mtROS	1.7	25	HIF⊣AMPK	1.3	10
HIF-1→noxROS	0.1	15	AMPK⊣AMPK	0.6	5
ROS→AMPK	2	20	AMPK⊣HIF-1	1.2	10
HIF-1→HIF-1	1.5	20	P53⊣HIF-1	0.9	5
ROS→HIF-1	1.1	20	miR200⊣ZEB1	0.9	5
SNIAL→ZEB1	2	20	miR145⊣ZEB1	0.9	5
HIF-1→ZEB1	0.4	15	P53⊣OCT4	1.6	20
ZEB1→ZEB1	0.7	20	miR145⊣OCT4	1.9	10
OCT4→OCT4	1	10	miR145⊣MDM2	1.6	15
P53→MDM2	1.2	20	SNIAL⊣SNAIL	0.1	15
P53→miR145	0.6	20	miR34⊣SNAIL	1.8	5
OCT4→miR200	1.9	20	OCT4⊣miR145	1.1	25
P53→miR200	2	10	ZEB1⊣miR145	1.9	5
P53→miR34	1.4	5	ZEB1⊣miR200	0.6	5
AMPK→P53	1	20	SNAIL⊣miR200	0.1	20
HIF-1→P53	1.5	15	SNAIL⊣miR34	1.9	5
AMPK⊣mrROS	0.1	25	ZEB1⊣miR34	0.9	25
HIF-1⊣mrROS	0.3	5	MDM2⊣P53	1.2	10
AMPK⊣noxROS	0.5	15			

Supplemental Reference

1. Kang X, Wang J, Li C (2019) Exposing the underlying relationship of cancer metastasis to metabolism and epithelial-mesenchymal transitions. iScience 21: 754–772.

2. Pastushenko I, Brisebarre A, Sifrim A, Fioramonti M, Revenco T, et al. (2018) Identification of the tumour transition states occurring during emt. Nature 556: 463.

3. Zhang J, Tian XJ, Zhang H, Teng Y, Li R, et al. (2014) Tgf-β–induced epithelial-to-mesenchymal transition proceeds through stepwise activation of multiple feedback loops. Science signaling 7: ra91–ra91.

4. Lu M, Jolly MK, Levine H, Onuchic JN, Ben-Jacob E (2013) Microrna-based regulation of epithelial–hybrid–mesenchymal fate determination. Proceedings of the National Academy of Sciences 110: 18144–18149.

5. Jia D, Jolly MK, Tripathi SC, Den Hollander P, Huang B, et al. (2017) Distinguishing mechanisms underlying emt tristability. Cancer Convergence 1: 2.

6. Jolly MK, Boareto M, Huang B, Jia D, Lu M, et al. (2015) Implications of the hybrid epithelial/mesenchymal phenotype in metastasis. Frontiers in Oncology 5: 155–155.

7. Shamir ER, Pappalardo E, Jorgens D, Coutinho K, Tsai W, et al. (2014) Twist1-induced dissemination preserves epithelial identity and requires e-cadherin. Journal of Cell Biology 204: 839–856.

8. Kang X, Li C (2020) Landscape inferred from gene expression data governs pluripotency in embryonic stem cells. Computational and Structural Biotechnology Journal 18: 366 – 374.

9. Li C, Wang J (2013) Quantifying cell fate decisions for differentiation and reprogramming of a human stem cell network: landscape and biological paths. PLOS Computational Biology 9.
